# First-in-human immunoPET imaging of HIV-1 infection using ^89^Zr-labeled VRC01 broadly neutralizing antibody

**DOI:** 10.1038/s41467-022-28727-5

**Published:** 2022-03-09

**Authors:** Denis R. Beckford-Vera, Robert R. Flavell, Youngho Seo, Enrique Martinez-Ortiz, Maya Aslam, Cassandra Thanh, Emily Fehrman, Marion Pardons, Shreya Kumar, Amelia N. Deitchman, Vahid Ravanfar, Brailee Schulte, I-Wei Katherine Wu, Tony Pan, Jacqueline D. Reeves, Christopher C. Nixon, Nikita S. Iyer, Leonel Torres, Sadie E. Munter, Tony Hyunh, Christos J. Petropoulos, Rebecca Hoh, Benjamin L. Franc, Lucio Gama, Richard A. Koup, John R. Mascola, Nicolas Chomont, Steven G. Deeks, Henry F. VanBrocklin, Timothy J. Henrich

**Affiliations:** 1 Department of Radiology and Biomedical Imaging, University of California San Francisco, San Francisco, CA USA; 2 Division of HIV, Infectious Diseases and Global Medicine, University of California San Francisco, San Francisco, CA USA; 3 Division of Experimental Medicine, University of California San Francisco, San Francisco, CA USA; 4 Department of Microbiology, Infectiology and Immunology, Centre de Recherche du CHUM, Université de Montréal, Montreal, QC Canada; 5 Department of Clinical Pharmacy, University of California, San Francisco, USA; 6 Monogram Biosciences, Inc., Laboratory Corporation of America, South San Francisco, San Francisco, USA; 7 Department of Radiology, Stanford University, Palo Alto, CA USA; 8 Vaccine Research Center, National Institute for Allergy and Infectious Diseases, National Institutes of Health, Bethesda, MD USA

**Keywords:** Translational research, HIV infections, HIV infections

## Abstract

A major obstacle to achieving long-term antiretroviral (ART) free remission or functional cure of HIV infection is the presence of persistently infected cells that establish a long-lived viral reservoir. HIV largely resides in anatomical regions that are inaccessible to routine sampling, however, and non-invasive methods to understand the longitudinal tissue-wide burden of HIV persistence are urgently needed. Positron emission tomography (PET) imaging is a promising strategy to identify and characterize the tissue-wide burden of HIV. Here, we assess the efficacy of using immunoPET imaging to characterize HIV reservoirs and identify anatomical foci of persistent viral transcriptional activity using a radiolabeled HIV Env-specific broadly neutralizing antibody, ^89^Zr-VRC01, in HIV-infected individuals with detectable viremia and on suppressive ART compared to uninfected controls (NCT03729752). We also assess the relationship between PET tracer uptake in tissues and timing of ART initiation and direct HIV protein expression in CD4 T cells obtained from lymph node biopsies. We observe significant increases in ^89^Zr-VRC01 uptake in various tissues (including lymph nodes and gut) in HIV-infected individuals with detectable viremia (*N* = 5) and on suppressive ART (*N* = 5) compared to uninfected controls (*N* = 5). Importantly, PET tracer uptake in inguinal lymph nodes in viremic and ART-suppressed participants significantly and positively correlates with HIV protein expression measured directly in tissue. Our strategy may allow non-invasive longitudinal characterization of residual HIV infection and lays the framework for the development of immunoPET imaging in a variety of other infectious diseases.

## Abstract (web summary)

Here, the authors apply positron emission tomography (PET) imaging to visualize HIV tissue-wide burden in infected individuals using a radiolabeled broadly neutralizing antibody, ^89^Zr-VRC01, and show that PET tracer lymph node uptake positively correlates with HIV protein levels measured directly from cells obtained from these tissues. This strategy may allow non-invasive characterization of residual HIV infection in the setting of therapeutic interventions.

## Introduction

Despite the overwhelming success of ART to achieve complete or near-complete HIV suppression, residual virus that integrates into host cell genomes prior to ART initiation persists indefinitely. A major obstacle to achieving long-term antiretroviral (ART) free remission or functional cure of HIV infection is the presence of these persistently infected cells that establish a long-lived viral reservoir^1–3^. HIV largely resides outside of the peripheral circulation^4,5^, and thus, numerous anatomical and lymphoid compartments that have the capacity to harbor HIV are inaccessible to routine sampling. As a result, there is a limited understanding of the tissue-wide burden of HIV infection and anatomical distribution of ongoing HIV-1 transcriptional activity, virion production, and viral replication in both untreated individuals and those on ART. Novel, non-invasive, in vivo methods are urgently needed to address this fundamental gap in knowledge. A positron emission tomography (PET)-based imaging approach using a radiolabeled SIV anti-gp120 monoclonal antibody has been applied to assess active infection in macaques^6,7^, but similar approaches in humans have yet to be successful.

The development of a PET-based approach to identify tissue with ongoing HIV translational activity and viral envelope production would provide valuable information into the pathogenesis and dynamics of the HIV reservoir, and facilitate the successful design and implementation of HIV eradication or immune-based therapeutic strategies^8^. As a result, we describe the development and application of a whole-body PET-magnetic resonance (MR) imaging approach to detect and anatomically localize regions of HIV infection in viremic and ART-suppressed people living with HIV. The objectives of the study are to determine the dosimetry and whole body distribution of the novel PET tracer, ^89^Zr-VRC01, in healthy volunteers and participants with HIV and evaluate uptake and tissue distribution of PET tracer in participants with HIV on ART. Here, we show that ^89^Zr-VRC01 uptake in various tissues (including lymph nodes and gut) is higher in HIV-infected individuals with detectable viremia and on suppressive ART compared to uninfected controls. Importantly, PET tracer uptake in inguinal lymph nodes in viremic and ART-suppressed participants significantly and positively correlates with HIV protein expression measured directly in tissue.

## Results

We prepared ^89^Zr-VRC01 and collected the requisite preclinical data to support the submission of an FDA IND application. Following FDA and local IRB approvals, we performed whole body PET/MR imaging to evaluate the radiation absorbed dosimetry in a cohort of uninfected controls, that also served as baseline comparators for distribution of ^89^Zr-VRC01 in viremic and ART suppressed participants. Whole body PET images were taken in five viremic and five ART-suppressed participants, with lymph node sampling in a subset of five individuals. As detailed below, uptake in tissues known to harbor HIV-infected cells were visualized in viremic and ART suppressed participants.

### Development of ^89^Zr-VRC01, a HIV-Specific PET Tracer

We obtained GMP grade VRC01, under a materials license agreement from the NIH Vaccine Research Center, for use in this first-in-human imaging study, as published clinical trials have demonstrated both safety and efficacy of delaying viral rebound following analytical treatment interruption^9–11^. VRC01 is a broadly neutralizing monoclonal antibody (bnAb) that interacts with contacts within the gp120 loop D, the CD4 binding loop, and the V5 region of the HIV-1 gp120 envelope protein^9,11–15^. Bioconjugation of GMP VRC01 with the bifunctional chelator DFO (desferoxamine)-isothiocyanate was performed using established radiochemistry methods and summarized in Fig. S1^16^. Zirconium-89 (half-life = 78.4 h, 23% positron emission) was then complexed to the DFO-bound VRC01. The longer half-life enables serial imaging over a week, providing ample time for mAb uptake and distribution in tissues with clearance from non-target tissues—all factors that may be critical to effective imaging of tissue HIV burden in vivo. Following conjugation, the ability of VRC01 to bind to HIV Env CD4 binding site RSC3 protein was evaluated. Over four separate experiments we determined the dissociation constant (*K*_d_) to be 5.21 ± 0.84 nM (Fig. S2); the *K*_d_ was similar to native VRC01 antibody (5.62 nM)^12^ indicating that radioisotope/chelate conjugation does not significantly alter VRC01 binding affinity.

### In vivo preclinical dosimetry studies

The standard of practice for first-in-human PET tracer studies for IND submission is to use murine models to generate organ and whole body effective radiation exposure (dosimetry) to estimate initial human tracer dosing. The dosimetry of ^89^Zr-VRC01 in five male and five female wild type Balb/C mice were studied by serial microPET/CT imaging over 5 days (Fig. S2). The radiation time activity curves in several organs were used to estimate the whole body and organ radiation absorbed dose (dosimetry) as shown in Table S1. The mouse dosimetry data was used to establish the human tracer dosing [approximately 1 mCi (37 MBq) intravenous (IV) injection] with a projected whole body radiation exposure of 12 mSv, which is considered to present minimal increased cancer risk. The liver was the organ with the largest absorbed dose in both the mouse dosimetry studies and the initial human data as below. The use of MRI in lieu of CT scanning for PET image attenuation correction and anatomic localization reduced cumulative radiation exposure from longitudinal imaging.

In preparation for exploratory investigational new drug (IND) submission, pre-release and post-release assays were performed on the manufactured ^89^Zr-VRC01 as shown in Table S2. These assessments included radiochemical purity using rTLC (radio-thin layer chromatography) and rad-SE-HPLC (radio-size exclusion high performance liquid chromatography) for identity and purity, visual observation for solution clarity, pH, endotoxin concentration, final filter integrity, ^89^Zr-VRC01 RSC3 binding, and sterility testing. Stability testing demonstrated that the dose was suitable for injection up to 24 h at room temperature. Tracer release criteria included radiochemical purity ≥90%, and our observed stability ranged from 97.4 to 99.4%. Importantly, immunoreactivity against HIV gp120 protein was maintained over this time with less than 20 nM binding affinity. The VRC INDs (IND 113611 and IND 126001) were cross referenced for the VRC01 pharmacokinetic and toxicologic information. The human imaging protocol and ^89^Zr-VRC01 manufacturing information was submitted to the FDA in the IND package for review. FDA approval was granted (IND 139652) for human use.

### Study cohort, participant selection, VRC01 susceptibility and demographic information

Upon local institutional review board and radiation safety committee approvals we enrolled and performed serial PET-MR imaging on 15 study participants, which included five viremic individuals (plasma HIV-1 RNA ranging from 3499 to 789,705 copies/mL), 5 ART-suppressed individuals (time on ART ranging from 105 to 8509 days on ART), and five uninfected control participants. These participants were selected in order to determine the performance of ^89^Zr-VRC01 PET imaging in the setting of a range of viral load measurements and duration of ART. The University of California San Francisco Committee on Human Research approved this clinical study and all participants provided written informed consent and underwent MRI screening prior to imaging. Demographic information, treatment histories, and virological and immune parameters for each participant are shown in Table 1.

**Table 1:** Participant demographics and HIV disease and immunologic status at the time of PET imaging study enrollment.

ID	Group	ART regimen	Days on ART^a^	Viral load^b^	CD4+ T cell count^c^	Lymph node sampling^d^
V1	Viremic	None	–	74,534	192	Y
V2	Viremic	None	–	19,872	243	Y
V3	Viremic	None	–	789,705	36	N
V4	Viremic	None	–	106,994	19	Y
V5	Viremic	None	–	3499	500	Y
A1	ART	ABC/3TC/DTG	105	ND^e^	578	Y
A2	ART	TAF/FTC/EVG/c	3150	ND	526	N
A3	ART	ABC/3TC/DTG	8509	ND	621	N
A4	ART	RPV/DTG	640	ND	650	N
A5	ART	TAF/FTC/EGV/DRV/c	6863	ND	527	N
C1	Control	–	–	–	1503	–
C2	Control	–	–	–	908	–
C3	Control	–	–	–	762	–
C4	Control	–	–	–	702	–
C5	Control	–	–	–	158	–

*ART* antiretroviral therapy, *ABC* abacavir, *DTG* dolutegravir, *3TC* lamivudine, *TAF* tenofovir alafenamide, *RPV* rilpivirine, *EVG* elvitegravir, *c* cobicistat, *DRV* darunavir.

^a^Continuous ART since last viremic episode.

^b^Plasma HIV-1 RNA copies/mL.

^c^Cells/mL.

^d^Participant provided additional consent to under inguinal lymph node needle biopsy.

^e^Plasma HIV-1 RNA not detected (limit of quantification 40 copies/mL).

The mean ages of uninfected control, viremic and ART-suppressed participants were 58, 52, and 53 years, respectively (Table 2). We made an effort to enroll females in this study, who were not pregnant or breastfeeding prior to or following tracer injection and PET imaging. Individuals were excluded if they had a history of systemic malignancy, recent opportunistic infections, systemic autoimmune or inflammatory disorders, or were taking concomitant immune modulating medications. An independent safety monitoring committee was formed to review any potential adverse events.

**Table 2:** Summary of age and sex of participants based on HIV diagnosis and antiretroviral treatment.

Group	% Male	Mean age in years (95% CI)
Viremic	80	52.2 (47.3–57.1)
ART	100	53.2 (32.5–74)
Uninfected control	80	58.4 (41.1–75.7)

*ART* antiretroviral therapy, *CI* confidence interval.

Although there are few known canonical mutations known to confer VRC01 resistance, we isolated HIV DNA from peripheral blood mononuclear cells (PBMC) and performed HIV-1 Env sequencing from HIV-infected participants to determine the absence of mutations in previously described regions associated with VRC01 binding (e.g., β23-loop V5 loop, loop D, and CD3 binding loop)^9,11,12,17^ that may inhibit mAb engagement with HIV envelope (Table S3). Samples were also tested using the PhenoSense mAB monoclonal antibody assay (Monogram Biosciences) in order to determine neutralization inhibitory concentration 50% (IC50) of psuedoviruses derived from participant PBMC HIV DNA modified from reported assays using viral RNA (MG-SF-VALD-VR0997: Validation Report: PhenoSense Monoclonal Antibody (mAb) Assay; Effective 10/21/2019.)^18^. Envelope amplification psuedovirus creation was successful for all five viremic participants and 2 of 5 ART-suppressed individuals. With the exception of one viremic participant (V1), IC50 ranged from 0.09 to 0.6 µg/mL and 100% of neutralization was able to be obtained at assay-defined drug concentrations (Fig. S3). Participant V1 had an IC of 16 µg/mL. IC50 for the HIV JR_CSF_ clone ranged from 0.14 to 0.18 µg/mL.

### In vivo immunoPET imaging of HIV-1 infection

PET and MR data were collected over a 25 cm axial field of view (bed position) from the head to mid thighs (six bed positions). Longitudinal PET-MR images were initially acquired at four time points following ^89^Zr-VRC01 injection (1–2, 4–6, 24, and 72 h) to determine the optimal timing of PET-MR imaging and to collect data for human dosimetry assessment. Dosimetry data was generated from analysis of a subset of (*N* = 6) participants as shown in Table S3. Our initial tracer dose was determined by extrapolating end the organ radiation exposure from mouse data (Table S1). Overall, the absorbed radiotracer dose was similar to the mouse model estimates (0.343 mGy/MBq) with the highest uptake noted in excretion organs such as liver, kidneys, and gallbladder wall, in addition to the heart wall (Table S1). Liver uptake was higher in humans (2.905 mGy/MBq) than mice (0.514 mGy/MBq), but critical organs including lens of eye/brain, reproductive organs, and hematopoietic tissue (i.e., bone marrow) all had similar or lower doses to what was observed in the mice. As a result, we did not make any modification to the administered dose.

To identify potential differences in ^89^Zr-VRC01 uptake between viremic and uninfected control participants, we initially enrolled four viremic and four uninfected individuals and performed serial PET-MR imaging over 3 days, as detailed above, following 18.5–37 MBq (0.5–1 mCi) tracer injection. Maximum and mean tracer standardized uptake values (SUVmax, SUVmean) were determined by 3-dimensional spherical gating on tissue regions of interest (ROI) using the OsiriX DICOM viewer software package (Pixmeo; Bernex Switzerland). ROI gating was performed blinded as to participant cohorts (i.e., viremic, ART suppressed, and control) and on the MRI images prior to fusion with PET uptake data in order to minimize region selection bias based on tracer uptake values. Minor adjustments were made to gating following PET data fusion in order to make sure that ROI did not overlap intravascular or intestinal intra-lumen signal.

SUVs are a function of the concentration of radiation activity within a ROI, the administered radiation dose, participant weight, timing of injection and tracer decay rates^19^, allowing cross-participant comparisons of PET activity. Ratios of the SUV within a tissue ROI to the blood pool in the aortic outflow tract were then calculated (SUV ratio; rSUV). SUVmax (or rSUVmax) is a measure of the maximum uptake in any tissue ROI voxel whereas SUVmean is an average of the gated 3-dimensional tissue area^19^. Both are used commonly in PET image analysis and are subject to various strengths and limitations. For example, SUVmax values change less with variations in ROI gate position or size, whereas SUVmean values provide information within an entire tissue area of interest. This method has the potential to minimize biasing factors such as tracer excretion rates between participants and has been shown in solid tumor FDG PET studies to improve prognostic and clinical value of imaging data^20–26^. rSUV values allow a direct measure of selective tracer uptake within tissues compared to circulating tracer and important given the long biological and radiological half-lives of ^89^Zr-VRC01.

**Fig. 1:**
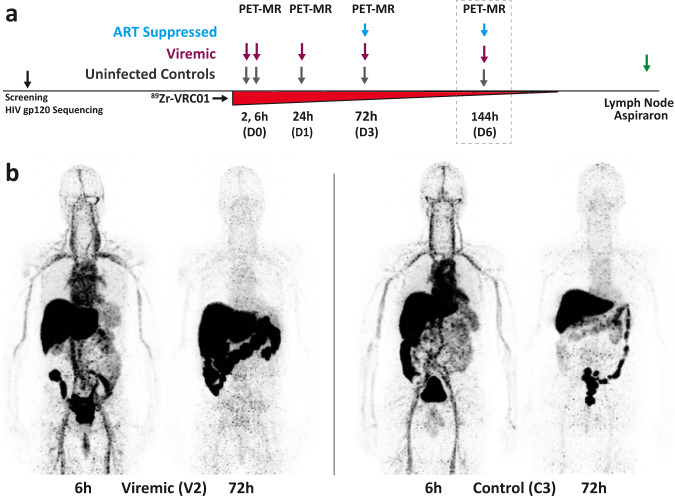
First-in-human immunoPET imaging of HIV persistence. A schematic of PET-MR imaging workflow including tracer administration and imaging time points is shown in **a**. Imaging on day 6 was limited to a subset of participants to determine if meaningful information could be obtained over longer periods of time various tissues of interest. Representative 3-dimensional maximum intensity projection images (MR attenuation corrected, but without MR overlay) of ^89^Zr-VRC01 uptake 6 and 72 h in a viremic (V2) and uninfected control (C3) participant are shown (**b**).

The differences in tissue uptake in viremics compared to uninfected controls were the greatest at ≥24 h after ^89^Zr-VRC01 administration for most tissues. We imaged all subsequent participants (one additional viremic and each control participant) and five ART-suppressed individuals at ~72 h (D3). In an exploratory sub-study in order to determine how long following tracer injection PET signal may be detected, we further imaged one viremic, one uninfected control and four ART-suppressed participants 144 h (6 days, D6) following ^89^Zr-VRC01 administration. Figure 1 shows a schematic of imaging workflow and representative 3-dimensional maximum intensity projection images (MR attenuation corrected, but without MR overlay) of ^89^Zr-VRC01 uptake 6 and 72 h in a viremic and uninfected control participant. The mass microdose (approximately 1 mg of VRC01 associated 37 MBq of ^89^Zr-VRC01) was well tolerated without any reported adverse clinical events of any grade, changes in vital signs or electrocardiogram readings.

Increased ^89^Zr-VRC01 uptake in lymphoid and gastrointestinal tissues in viremic and ART-suppressed participants compared with uninfected individuals.

**Fig. 2:**
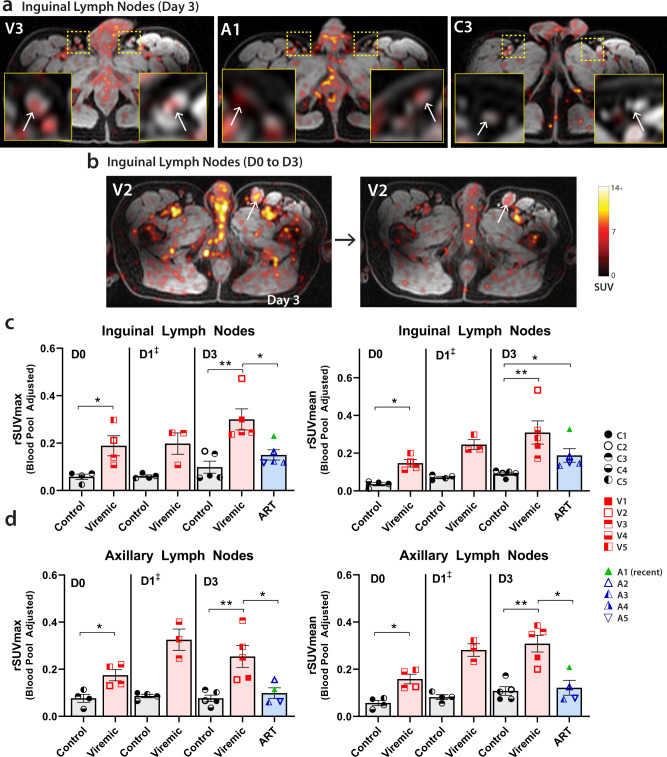
^89^Zr-VRC01 uptake is preferentially increased in participants with HIV compared with uninfected controls. **a** Representative axial PET-MR images with inset magnification of bilateral inguinal lymph nodes on imaging day 3 for a viremic (V), ART-suppressed (A) and uninfected control (C). **b** Axial image of persistent inguinal lymph node ^89^Zr-VRC01 uptake in inguinal lymph nodes on imaging day 0 (6 h post injection), and imaging day 3 (72 h post injection) (**b**). rSUVmax and rSUV mean values for **c** inguinal and **d** axillary lymph node uptake averaged across bilateral nodes at imaging day 0, day 1, and day 3. Participant A1 (green triangle) started ART approximately 3 months prior PET imaging (early treatment initiation). ART suppressed participants were imaged starting on day 3 as per study protocol. SUV standardized uptake value, rSUV blood pool-adjusted ^89^Zr-VRC01 standardized uptake value (tissue to blood pool SUV ratio); *P* values <0.05 and <0.01 represented by * and **, respectively from two-sided non-parametric analyses (Mann–Whitney tests for analyses involving two groups and Kruskal–Wallis tests for analyses involving three groups). Mean and standard error bars are shown. Control *N* = 4 on day 0 and day 1, viremic case *N* = 4 and 3 on day 0 and day 1, respectively (^‡^statistical analyses not performed on day 1 as one viremic participant missed this visit). Control and viremic and ART case *N* = 5 on day 3. Source data are provided as a Source Data file.

We measured uptake in bilateral axillary and inguinal lymph nodes in participants at each imaging time point. rSUVmax and rSUVmean values were calculated based on averages of SUV of at least two nodes in each region. As above, ROI gating was performed on MR images alone prior to PET data import and overlay in order to minimize potential reviewer bias. As shown in Fig. 2, significantly higher inguinal and axillary lymph node rSUVmax and rSUVmean were observed in viremic compared to uninfected participants at all time points. Fold changes in lymph node rSUVmax and rSUVmean between participants with HIV and uninfected controls are shown in Table S4. Importantly, inguinal lymph node rSUVmean values were also significantly higher in ART-suppressed participants compared with controls (fold changes 2.47–3.08) across all timepoints, but lower than values observed in viremic individuals on imaging day 3 (fold change 1.99). Of note, lymph node rSUV were also observed to be higher in viremic compared with uninfected participants on imaging day 1 (Fig. 2), but one viremic participant did not have PET-MR imaging at this timepoint, so no statistical measures were applied to this imaging time point as less than four data points were available. Axial PET-MR images from inguinal/pelvic regions from all participants and lymph nodes in representative participants on imaging day 3 are shown in Figs. S4 and S5, respectively.

Background rSUVs in control participants were very low at all time points with exception of bowel intraluminal stool signal as detailed below, and more tightly clustered, suggesting low non-specific tissue uptake of radiolabeled antibody. In contrast, we observed an increase in rSUV in HIV-infected individuals over subsequent imaging time points suggesting that PET tracer uptake is more directly related to HIV infection and accumulates in tissues that are considered as major sites of HIV persistence over time. Other lymph node chains are of interest in HIV persistence, especially in the mesentery which may harbor large numbers of virus-infected cells. However, the PET-MR imaging technique involved led to peristalsis and gut-motion artifact which made gating challenging.

**Fig. 3:**
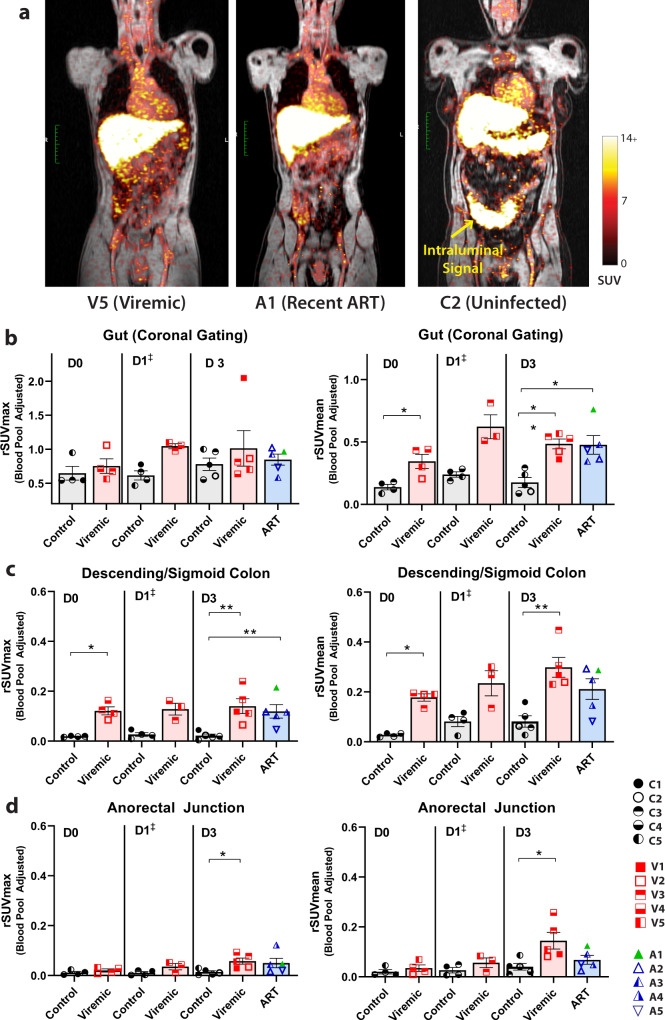
^89^Zr-VRC01 uptake is preferentially increased in gut tissue in participants with HIV compared with uninfected volunteers up to three days following injection. **a** Representative coronal PET-MR images showing increased gut tracer uptake in participants with HIV on or off ART. First, gating was performed on coronal images across multiple bowel loops excluding obvious intraluminal stool signal which likely represented HIV non-specific ^89^Zr-VRC01 excretion (**b**). rSUV values for sigmoid/descending colon wall (**c**) and anorectal tissue (**d**) were obtained by gating exclusively on multiple sections of bowel wall to exclude intraluminal signal. SUV standardized uptake value; rSUV blood pool-adjusted ^89^Zr-VRC01 standardized uptake value (tissue to blood pool SUV ratio); *P* values <0.05 and <0.01 represented by * and **, respectively from two-sided non-parametric analyses (Mann–Whitney tests for analyses involving two groups and Kruskal–Wallis tests for analyses involving three groups). Mean and standard error bars are shown. Control *N* = 4 on day 0 and day 1, viremic case *N* = 4 and 3 on day 0 and day 1, respectively (^‡^statistical analyses not performed on day 1 as one viremic participant missed this visit). Control and viremic and ART case *N* = 5 on day 3. Source data are provided as a Source Data file.

Gut-associated lymphoid tissue is a major anatomical site of HIV replication, CD4+ T cell depletion in acute and early infection, and a major site of HIV persistence on ART.^27–31^ Significantly increased ^89^Zr-VRC01 rSUVmean uptake was observed in both viremic and ART-suppressed individuals in various gut tissues as shown in Fig. 3. Fold changes in gut rSUVmax and rSUVmean between participants with HIV and uninfected controls are shown in Table S4. Importantly, fold changes in adjusted SUV between participants with HIV, including those on ART) and uninfected participants ranged from 2.59 to 6.53 in the descending colon and 1.69 to 4.1 in the anorectal area. Of note, gating on bowel tissue was challenging as we observed intermittent intraluminal collection of tracer which varied over time for all participants, including uninfected individuals. As this signal does not represent HIV-specific tissue tracer uptake, gating was initially performed in gut bowel loop regions from coronal images without obvious intraluminal signal as shown in Fig. 3, as previously reported in SIV non-human primate immunoPET imaging^6^. SUVmean (or rSUVmean) may be more robust for this relatively macro-scale gut analyses, as the presence of even small amounts of residual intraluminal signal can influence maximum uptake values, despite best efforts to exclude these areas in gated ROI. In addition, intraluminal tracer excretion levels may also be much higher than the circulating blood pool.

To further determine the capacity for ^89^Zr-VRC01 to identify differences in gut tissue between participant cohorts, we performed ROI analysis on two additional regions, descending/sigmoid colon and anorectal junction. SUV were averaged from two non-contiguous ROI colon or anorectal wall segments away from stool-filled loops and taking care to avoid intraluminal overlap in gating. We observed significantly increased rSUVmax and rSUVmean values from the descending/sigmoid colon wall tissue in viremic versus control participants at both imaging D0 and D3 timepoints, and significantly increased rSUVmean values in ART suppressed participants compared to uninfected participants at these timepoints (Fig. 3). Tracer uptake levels were lower in anorectal tissue, although we observed a significant increase in signal (rSUVmax and rSUVmean) in viremic compared with uninfected participants on D3 (Fig. 3).

In order to determine if meaningful information could be obtained over longer periods of time in lymphoid tissues of interest, PET-MR imaging was performed 6 days following tracer administration in all ART suppressed individuals as well as in a representative viremic and an uninfected control participant. We observed overall increased rSUVmax in lymphoid tissues (inguinal lymph nodes, axillary lymph nodes, gut) in the viremic individuals. and to a lesser extent, ART-suppressed participants, compared with the representative control participant as shown in Fig. S6.

### ^89^Zr-VRC01 uptake in other tissues

**Fig. 4:**
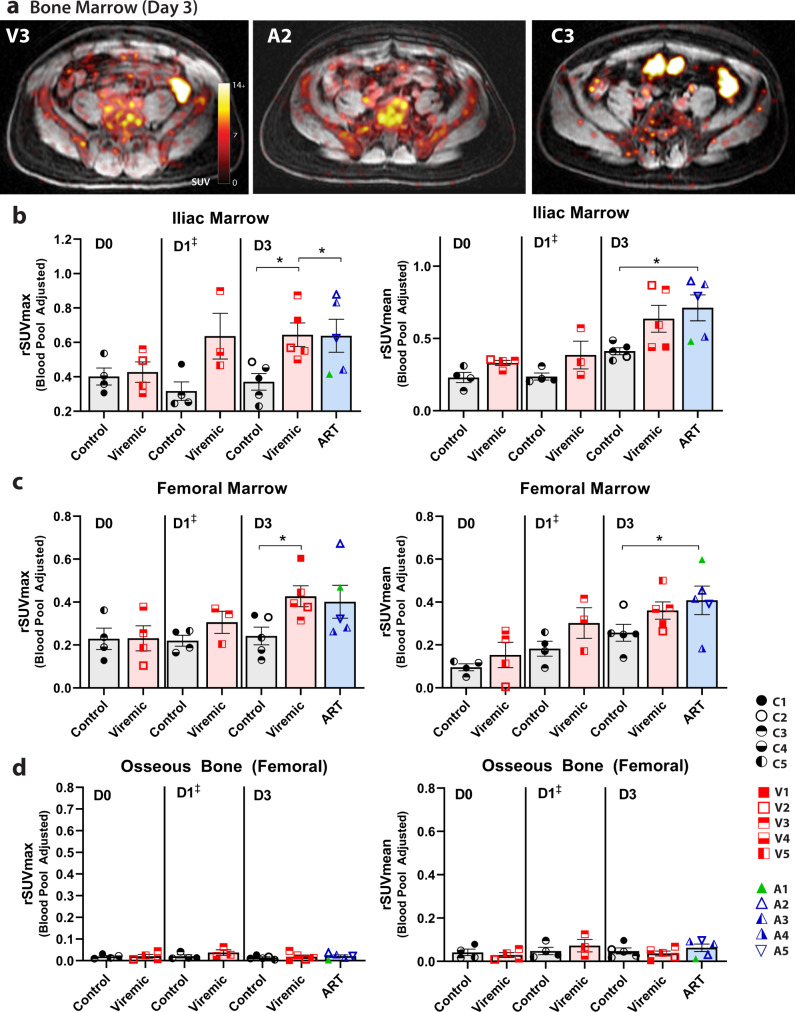
^89^Zr-VRC01 uptake is preferentially increased in iliac and femoral bone marrow in participants with HIV compared with uninfected volunteers 3 days following injection. **a** Representative axial PET-MR images showing tracer uptake in axial marrow in representative viremic, ART suppressed and uninfected participants. rSUVmax and rSUV mean values for **b** iliac and **c** femoral shaft bone marrow ^89^Zr-VRC01 uptake from 3-dimensional gating at imaging day 0, day 1, and day 3. Minimal uptake was observed in osseus (femoral shaft) bone at any time point in all cohorts (**d**). SUV standardized uptake value; rSUV blood pool-adjusted ^89^Zr-VRC01 standardized uptake value (tissue to blood pool SUV ratio); *P* values <0.05 and <0.01 represented by * and **, respectively from two-sided non-parametric analyses (Mann–Whitney tests for analyses involving two groups and Kruskal–Wallis tests for analyses involving three groups). Mean and standard error bars are shown. Control *N* = 4 on day 0 and day 1, viremic case *N* = 4 and 3 on day 0 and day 1, respectively (^‡^statistical analyses not performed on day 1 as one viremic participant missed this visit). Control and viremic and ART case *N* = 5 on day 3. Source data are provided as a Source Data file.

We observed unexpected prominent ^89^Zr-VRC01 uptake in bone marrow over time, which was higher than in uninfected control participants. Non-osseous axial (iliac) and femoral bone marrow uptake of ^89^Zr-VRC01 was higher in viremic and ART-suppressed participants compared with uninfected individuals as shown in Fig. 4. Fold changes in iliac and femoral bone marrow rSUVmax and rSUVmean between participants with HIV and uninfected controls are shown in Table 2. Unlike in lymph node and gut, however, a wider range of uptake values were observed in viremic and ART-suppressed individuals in bone marrow. We have not confirmed if the tracer uptake represents a HIV-specific engagement or due to increased blood flow, cellular hypermetabolism and non-specific tracer deposition, but these results indicate that further human studies of bone marrow are justified. While not traditionally considered a major site of HIV persistence, given a paucity of studies and difficulties with obtaining tissue, recent humanized mouse and non-human primate research have shown that marrow may be an important site of viral persistence involving both myeloid cells and lymphocytes^32,33^. Little to no tracer uptake was observed in femoral osseus bone (Fig. 4).

**Fig. 5:**
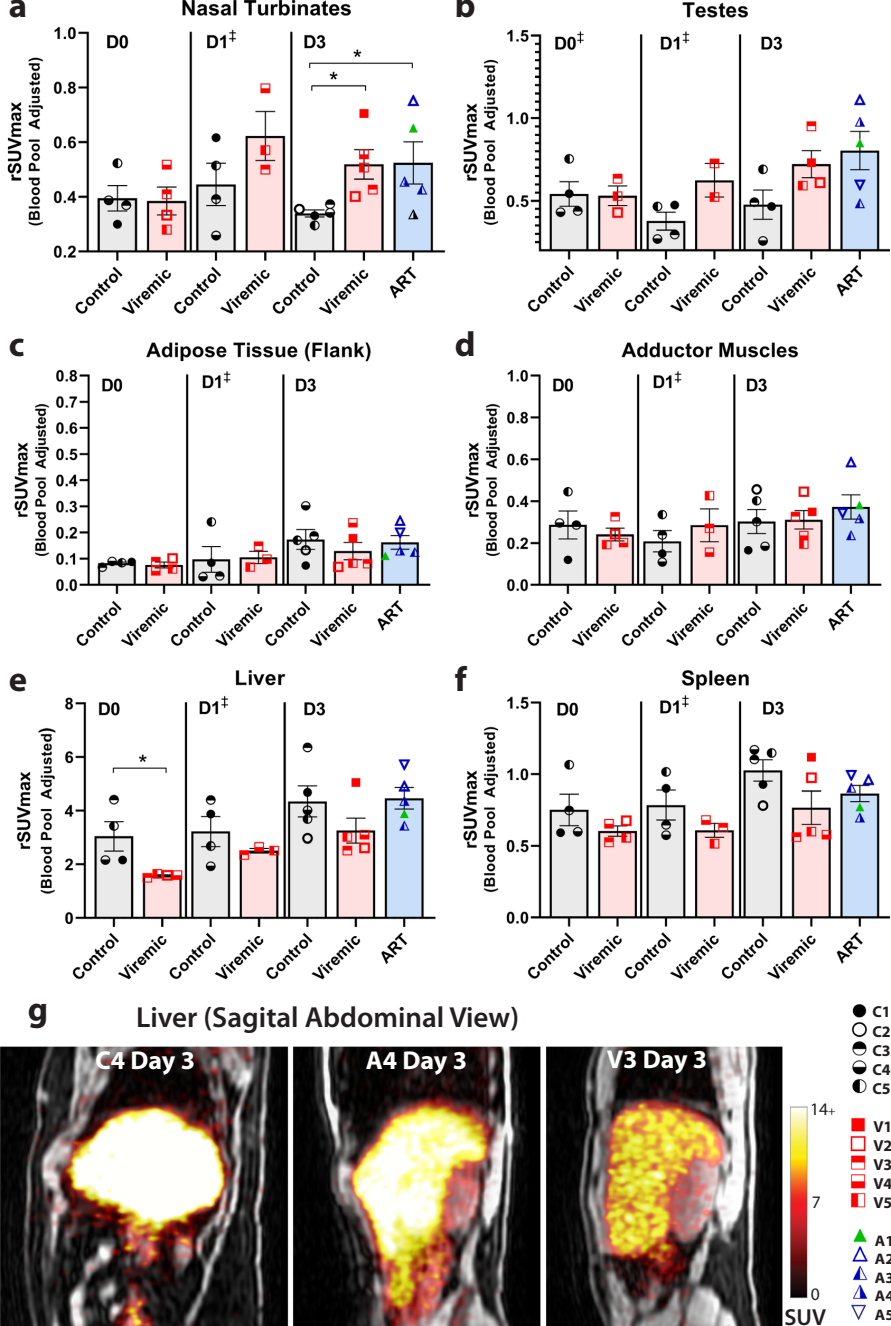
^89^Zr-VRC01 uptake in various tissues. rSUVmax values for **a** nasal turbinates, **b** testes, **c** adipose tissue (right flank), **d** adductor muscles (thigh), **e** liver and **f** spleen from 3-dimensional gating at imaging day 0, day 1, and day 3 are shown. Representative coronal PET-MR images showing tracer uptake liver as shown in **g**. ^89^Zr-VRC01 uptake was higher in viremic and ART-suppressed participants compared with uninfected controls on imaging Day 3. In contrast, tracer uptake in the liver was higher in uninfected controls (D0) may represents decreased lymphoid tissue and bone marrow distribution compared with participants with HIV. SUV standardized uptake value; rSUV blood pool-adjusted ^89^Zr-VRC01 standardized uptake value (tissue to blood pool SUV ratio); *P* values <0.05 and <0.01 represented by * and **, respectively from two-sided non-parametric analyses (Mann–Whitney tests for analyses involving two groups and Kruskal–Wallis tests for analyses involving three groups). Mean and standard error bars are shown. Control *N* = 4 on day 0 and day 1, viremic case *N* = 4 and 3 on day 0 and day 1, respectively (^‡^statistical analyses not performed on day 1 as one viremic participant missed this visit). Control and viremic and ART case *N* = 5 on day 3. Source data are provided as a Source Data file.

The prior primate SIV immunoPET study demonstrated increased tracer uptake in nasopharyngeal associated lymphoid tissue (NALT)^6^, and we also observed differences in ^89^Zr-VRC01 rSUVmax uptake between HIV-infected, ART-suppressed, and uninfected participants in the nasal turbinates as shown in Fig. 5. We also studied other tissues that may be important sites of HIV persistence that are difficult to obtain in routine clinical studies, including testes, brain, and spleen. As shown in Fig. 5, we observed non-significant differences in ^89^Zr-VRC01 uptake between HIV-infected and uninfected participants in testes, although background signal was more variable (rSUVmax values shown; complete ROI data provided in accompanying supplementary materials). Unfortunately, no discernible ^89^Zr-VRC01 uptake was observed in brain tissue of any participant, regardless of HIV infection or treatment regimen. Signal was observed in penetrating vessels, but not within the parenchyma, suggesting that the microdose of ^89^Zr-VRC01 administered does not cross the blood-brain barrier in significant quantities corroborating prior SIV immunoPET imaging studies^6,7^. Interestingly, there was a trend to lower rSUVmax in both viremic and ART-suppressed participants in the spleen and lower maximum uptake in the liver in compared with uninfected individuals. Liver is a site of tracer metabolism and lower levels may represent more antibody distributed throughout systemic tissues (e.g., gut, lymph nodes, bone marrow. etc.) in HIV-infected participants. In order to help determine if increased ^89^Zr-VRC01 uptake is specific to tissues known to harbor HIV (e.g., gut, lymph nodes), we include ROI such as adipose tissue (right flank) and adductor muscles and did not identify significant differences in overall low level rSUV uptake values between participant cohorts (Fig. 5).

### Relationship between ^89^Zr-VRC01 uptake and timing of ART initiation

**Fig. 6:**
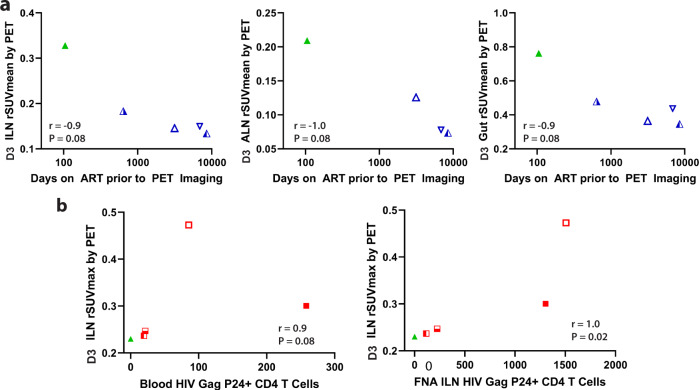
Associations between time on ART, HIV p24 protein expression in lymph node CD4 T cells obtained by tissue biopsy, and tracer uptake in various anatomical regions. Correlations between ^89^Zr-VRC01 rSUVmean in lymph node and gut tissues on imaging day 3 compared with days on ART for viral suppressed participants are shown in **a**. **b** Correlations between ^89^Zr-VRC01 rSUVmax/mean and the frequency of blood and inguinal lymph node HIV Gag P24+ CD4+ T cells as measured by flow cytometry. *r* and *P* values represent results from Spearman correlation analyses. Overall, inverse associations between tracer uptake and ART duration and a significant positive correlation between inguinal lymph node ^89^Zr-VRC01 uptake and direct measures of HIV protein expression in CD4 T cells from inguinal lymph node sampling were observed. Source data are provided as a Source Data file.

In order to better understand the relationship between ART use and PET signal in various tissue ROI, we correlated the number of days on suppression ART with rSUV uptake on imaging day 3, when ART-suppressed individuals were imaged. Interestingly, inguinal lymph node, axially lymph node and gut rSUVmean decreased with increased time on ART as shown in Fig. 6a. Spearman correlation coefficients ranged from −0.9 to −1, although with borderline significance (all *P* = 0.08) likely due to limited sample size. Further investigation on the relationship between timing of ART and tissue tracer uptake in larger cohorts are warranted.

### Inguinal lymph node HIV p24 levels correlate with ^89^Zr-VRC01 uptake in viremic and ART-suppressed participants

Correlation analyses in all HIV-infected participants revealed significant positive associations between inguinal and axially rSUVmax at imaging day 3 and plasma HIV-1 RNA levels (*r* = 0.78 and 0.83, respectively, both *P* = 0.02), but not uptake in other tissues or with rSUVmean (all *P* > 0.09). However, in order to help discern whether ^89^Zr-VRC01 uptake is a result of specific HIV protein interactions rather than non-specific uptake in inflamed tissues, we obtained consent from five participants (four viremic and one recently ART-suppressed) to obtain inguinal lymph node tissue from multiple needle aspirates. The frequency of CD4+ T cells expressing the HIV-1 p24 protein was determined by HIV-Flow^34^ in lymph node and matched blood cells. Whereas the number of participants was small, we identified a significant positive correlation between p24 levels measured directly from tissue and inguinal lymph node rSUVmax as determined by PET-MR imaging (*P* = 0.02; Fig. 6b). In contrast, the correlation between blood CD4 HIV-1 p24 levels and uptake values was less clear, with the caveat that sample size was small and analysis may have been influenced by a single outlier. Further, there were no significant positive correlations between inguinal lymph node HIV- 1 p24 levels and PET uptake in other tissues (all *P* > 0.1), suggesting a close relationship between specific local tissue HIV burden and tracer uptake.

## Discussion

We present the first in-human immunoPET imaging studies demonstrating uptake in viremic and ART-suppressed participants compared to uninfected individuals, even up to 6 days following a single injection of ^89^Zr-VRC01. Zirconium-89 was chosen for VRC01 imaging given that its >3-day half-life matches the biological half-life of mAbs and offers the opportunity to develop suitable image contrast with clearance from non-target tissue. While several mAbs have been labeled with copper-64 (12.7 h half-life)^6,7^, the imaging time is less than 2 days and may not be long enough to identify regions with lower target antigen concentrations.

Although our samples size was limited to five participants in each comparator group, we demonstrate that ^89^Zr-VRC01 uptake was significantly increased in various lymphoid and other tissues in HIV-infected individuals with detectable viremia and, to a somewhat lesser extent, on suppressive ART compared to uninfected control participants. Importantly, PET tracer uptake in inguinal lymph nodes from viremic and ART-suppressed participants who underwent lymph node sampling significantly and positively correlated with intracellular HIV protein expression. Besides, lower tracer uptake was observed in ART-suppressed individuals on longer-term ART. Together, these data suggest that PET tracer uptake may be associated with tissue HIV burden and has the potential to play an important role in HIV persistence studies and viral eradication efforts. A recently reported study by McMahon and colleagues^35^ evaluated a copper-64 (12.7 h half-life) labeled broadly neutralizing antibody, ^64^Cu-3BC117, in uninfected controls, viremic and aviremic HIV patients. They reported on the imaging and dosimetry which they found to be similar among all three cohorts. However, there was no elevated tracer accumulation noted in lymphoid tissue or the GI tract in the HIV patients. The shorter half-life of copper-64 or differences in antibody properties may account for the disparity with the study reported herein.

The largest differences between HIV-infected and uninfected participants was observed in lymphoid and various gut tissues which is consistent with prior non-human primate SIV immunoPET imaging studies and consistent with known major sites of HIV persistence^4,6,7,34,36^. However, we also observed increased ^89^Zr-VRC01 signal in both viremic and ART-suppressed participants in other anatomical sites such as bone marrow and the nasal turbinates. Splenic uptake of ^89^Zr-VRC01 was not increased in participants with HIV, as observed in the SIV study, which may be due to differences in radiotracer and the vascular nature of this organ impacting the observed SUV uptake ratios between the tissue and blood pool. Although it has yet to be shown that PET signal directly correlates with HIV Env expression in these tissues, our observations raise questions about the importance of these often-neglected anatomic sites of potential long-term HIV persistence. Interestingly, differences in tracer uptake between ART-suppressed and viremic participants were not observed in some of these tissues, which may reflect either a more “active” reservoir or require larger numbers of participants to observe more subtle differences. Combining PET-based imaging approaches and direct tissue studies of HIV persistence has the potential to expand the understanding of the whole-body burden and activity of HIV across the body, including other mucosal sites and help evaluate responses to HIV curative interventions^7^.

This study has several limitations inherent with first-in-human imaging using a novel tracer to identify low-level, persistent HIV Env gp120 expression. Overall, the participant cohorts were limited in size, with variability in plasma HIV-1 RNA copies or time on ART. For this proof-of-concept study, we chose participants with a range of such factors to understand the performance of ^89^Zr-VRC01 across a range of HIV disease phenotypes. We also observed some heterogeneity in tracer uptake between contralateral lymph nodes in specific areas of interest (e.g., inguinal, axillary) within participants. As a result, rSUV values were averaged across individual nodes within these areas. However, intra-participant heterogeneity of HIV protein expression within various tissues would be expected. Other lymph node chains of interest in HIV persistence, such as in the mesentery, were difficult to consistently identify and gate due to gut peristalsis and other motion artifacts with the PET-MR imaging approach. Improved imaging acquisition methods and more sensitive PET scanners may allow more detailed analysis of these important tissues.

ImmunoPET studies may involve administration of unlabeled (“cold”) antibody to block potential non-specific binding, thereby increasing signal-to-noise ratios. However, many of these studies involve radiotracers that target receptors that are ubiquitous on many cells and cell types despite being relatively upregulated in diseased tissues of interest^37–39^. HIV-1 gp120 expression is expected to be low, especially in ART-suppressed individuals, and the use of cold antibody runs the serious risk of blocking true signal as well as non-specific uptake. As a result, we chose not to use this strategy for initial first-in-human proof-of-concept studies. As above, we identified very low and tightly clustered background tracer uptake in lymphoid and other tissues in uninfected control participants. There is also a range of neutralization capacity of VRC01 to various in vivo HIV sequence variants across the population. Whereas variations within avidity of VRC01 binding to participants’ virus may bias correlations between HIV burden and tracer uptake, protein-tracer binding required for PET visualization may not require efficient viral neutralization. Larger studies incorporating direct measures of antibody binding and avidity are needed. One viremic participant demonstrated reduced, but not absent, neutralization in the PhenoSense mAb assay, but also had a relatively high viral load (>70,000 copies/mL) and further studies will be needed to more fully understand the impact of neutralization and antibody binding affinity with imaging correlates.

We observed differences in PET tracer uptake between HIV infected and uninfected individuals in lymphoid tissues up to 6 days following a single administration of ^89^Zr-VRC01. In an exploratory study of tracer uptake 6 days after injection also showed higher rSUV values in various lymph node and gut ROIs, although the limited sample size makes it difficult to make conclusions about detecting differences between ART-suppressed and viremic individuals compared with uninfected participants. Nonetheless, the data suggest that further studies using highly sensitive PET scanners over longer periods of time are warranted to better understand non-specific tracer uptake and specificity in various tissues. Using long-acting radioisotopes such as ^89^Zr may be important to allow sufficient time for antibody distribution and binding within various tissues and allow for serial imaging during clinical interventions in real time. ^89^Zr-VRC01 uptake in many tissue was lower than the blood pool, in which levels remained relatively high over time leading to ratio SUV values to be less than one. Nonetheless, differences in uptake and uptake ratios were identified in tissues across comparator groups. In addition, scattered, punctate foci of positron emission are observed in the 2-dimensional images. These may represent penetrating blood vessels or other non-specific background noise. These foci are usually not observed in contiguous planes and therefore do not factor greatly in the 3-dimensional ROI analyses employed. These small foci are also unlikely to have a major impact on mean SUVs and we observed similar trends in tissue in both mean and maximum SUV analyses.

HIV-1 gp120 is metastable and challenging to characterize and quantify both in vivo and in vitro^40–42^; stable expression of gp120 on the surface on CD4+ T or other cells in ART-suppressed participants is expected to be low. This is a potential limitation to all immunoPET approaches using gp120-specfic antibodies, regardless of their ability to neutralize virus in vivo or ex vivo. As a result, it is possible that the observed uptake of anti-gp120 bNAb represents binding to budding viral particles or recently released, local tissue-resident virions rather than stable surface expression of protein on cell surfaces. As a result, ^89^Zr-VRC01 PET signal may represent, at least in part, anatomical loci of persistent productive infection despite otherwise stable ART use. For example, in situ hybridization studies have shown that there is ongoing active HIV particle formation in primary and secondary lymphoid structures under the cover of ART, which supports this hypothesis^4^. On the other hand, there may be residual HIV gp120 proteins in tissues without active viral production or replication binding to bNAb. Nonetheless, we observed a significant correlation between lymph node intracellular HIV P24 protein and ^89^Zr-VRC01 uptake in this pilot study.

Overall, HIV-specific PET imaging strategies are showing promise to have the capacity to determine the spatial and temporal dynamics of HIV persistence. Non-invasive PET imaging techniques can be applied to a variety of ongoing or planned HIV clinical trials and may be particularly useful in identifying anatomical regions of viral recrudescence during analytical treatment interruption or viral reactivation during latency reversal. ImmunoPET approaches also have the potential to be used to characterize whole-body HIV burden over time and longitudinal responses to HIV therapeutic or curative studies. Larger immunoPET investigations including parallel direct tissue sampling are therefore warranted, and have the further potential to identify anatomical areas that have historically received less attention but may play important roles in HIV persistence. Finally, our study lays the framework for the development and implementation of immunoPET imaging in a wide variety of other infectious diseases.

This study incorporated ^89^Zr-VRC01 with a functional Fc domain, and it is possible that at least some of the increased signal in HIV-infected individuals represents non-specific uptake in areas of increased tissue inflammation (which is often closely related to the degree of HIV replication within the tissue) or immune complex formation and subsequent tissue deposition. Further studies involving fragment antigen binding (FAb) versions of broadly neutralizing antibodies are certainly warranted, but at the time of this investigation, none have been approved for human use, which was a requisite for our in vivo study. Nonetheless, the prior primate SIV immunoPET study used an antibody modified with a modified linear poly(ethylene glycol) to minimize Fc engagement and reported similar low background levels in uninfected controls^6^ as we observed in our study. Another option to reduce potential non-specific interactions includes in vivo administration of pooled immunoglobulin. However, we observed very tight clustering with predictable low levels of tissue tracer uptake and rSUV in the uninfected control participants suggesting background noise is predictably low in major tissue of interest. Moreover, we observed a close association between inguinal lymph node HIV p24 protein expression and ^89^Zr-VRC01 uptake in the same anatomical location suggesting the tracer specificity. Further studies in longitudinal cohorts with direct access to a wide range of tissues are needed to fully validate the relationship between PET signal and local HIV burden.

The rSUV tissue to blood ratios were, for the most part, less than one, suggesting that tissue signal was lower than blood signal across the comparator cohorts. However, tissue levels of mAbs are expected to have blood levels higher than tissue which distribute to tissue in proportion of increased target issue binding (relative to binding to circulating target in the blood)^43^. As above, mAb target (i.e., HIV envelope) expression is expected to be much lower in our study participants (and in particular in ART-suppressed individuals or people without HIV) compared to those participants in prior immunoPET studies that involve targets with much higher levels of cell-surface or tissue protein expression^7,44^. Some differences in uptake between tissues were subtle and required careful gating on un-rendered series of axial images with MR overlay for gating of structures such as individual lymph nodes to avoid potential overlap with intravascular signal. Gating was performed blinded to participant cohort prior to merging with PET datasets to minimize potential ROI selection biases. Regardless of these limitations, our study provides novel immunoPET imaging methods that may be adapted to a wide variety of studies involving low-level target expression.

## Methods

### Study cohort

The primary objectives of the study was to determine the dosimetry and whole body distribution of ^89^Zr-VRC01 in health volunteers and participants with HIV. The secondary objective was to evaluate uptake and tissue distribution of PET tracer in participants with HIV on ART compared with uninfected individuals. Fifteen participants 18 years of age or older were recruited for this imaging study, including HIV-infected participants on continuous suppressive ART with viral load <40 RNA copies/mL prior to imaging and within 12 months of study entry or detectable plasma HIV RNA >1000 copies per/mL, and uninfected controls. In addition, HIV+ participants had HIV-1 envelope RNA or DNA consensus sequences from peripheral blood suggestive of VRC01 binding activity (HIV infected participants only). This study represents initial data from an ongoing protocol after five participants were enrolled in each of three comparator groups (HIV on ART, HIV not on ART and uninfected controls. The study protocol defined the initial analysis stage of the study to include up to 18 individuals. Participants were excluded if they had any contraindications to MRI (e.g., permanent pacemaker, implantable metallic device/ prosthetic, aneurysm clip, non-removable piercing, or severe claustrophobia), underwent prior research study involving radiation within 6 months of enrollment, pregnant or breastfeeding, screening absolute neutrophil count <1000 cells/mm^3^, platelet count <70,000 cells/mm^3^, hemoglobin <8 mg/dL, estimated creatinine clearance <40 mL/min, aspartate aminotransferase >100 units/L, alanine aminotransferase >100 units/L, recent serious illness requiring hospitalization or parental antibiotics, or a current HIV-related opportunistic infection. A safety monitoring committee was established to evaluate safety of ^89^Zr-VRC01 at regularly scheduled intervals and if there were any grade 3 or greater adverse events. These data included participant reported adverse events (evaluated during PET imaging visits and by telephone follow up), ECG and vital sign monitoring. Participants were co-enrolled in the HIV SCOPE study for additional collection of blood and tissue for measures of HIV persistence as below.

### Institutional and regulatory approvals

The US FDA approved the use of ^89^Zr-VRC01 under IND 139652. The UCSF Committee on Human Research Institutional Review Board and the UCSF Radiation Safety Committee approved the study. written informed consent was obtained from each participant prior to study entry. ClinicalTrials.gov: NCT03729752. All animals were handled under UCSF IACUC approved protocols.

### Conjugation of p-isothiocyanatobenzyl-deferoxamine (DFO) with VRC01

DFO was conjugated to VRC01 following procedures previously described in the literature^45–48^. VRC01 was mixed with 5-fold molar excess of DFO in 0.1 M NaHCO_3_-Na_2_CO_3_ pH 9.0 for 45 min at 37.0 °C and purified by size-exclusion using a PD-10 desalting column and sodium acetate 0.25 M as a mobile phase. The concentration of DFO-VRC01 was determined by spectrophotometry using a Nanodrop. The number of DFO bound to one molecule of VRC01 was determined by isotopic dilution assay. The final compound was characterized by size-exclusion high performance liquid chromatography (SE-HPLC) and instant thin layer chromatography (iTLC)^45–48^. The conjugate was kept at 4 °C for up to 3 weeks.

### Radiolabeling of DFO-VRC01 with ^89^Zr

To an eppendorf vial containing 20 µL of deionized water, an aliquot of ^89^Zr-oxalate (2–6 mCi, 5–30 µL) was mixed with equal volume of 1 M sodium carbonate buffer pH 9.0 and let it to rest for 2 min. A solution of 1 M ammonium acetate 1 M (800 µL) and DFO-VRC01 conjugate in sodium acetate 0.25 M (1–3 mg) was added to the vial containing neutralized ^89^Zr. The solution was incubated at room temperature for 30 min to 1 h. ^89^Zr-DFO-VRC01 (^89^Zr-VRC01) was purified from free ^89^Zr using a PD-10 desalting column and a solution containing 5 mg/mL gentisic acid in 0.25 M sodium acetate pH 5.5. Fractions of 1 mL were collected and the fractions containing ^89^Zr-VRC01 were pooled together and analyzed. SE-HPLC and iTLC were used to determine chemical and radiochemical purity. The stability of the preparation was assessed 24 h post-preparation.

### ^89^Zr-VRC01 binding

^89^Zr-VRC01 binding affinity was assessed by measuring the interaction of ^89^Zr-VRC01 and Resurfaced Stabilized Core (RSC3) protein obtained by the NIH AIDS Reagent Program. RSC3 is a protein comprised of the CD4 binding site of the gp120 with the other HIV antigenic regions removed^49^. A 96-well ELISA plate was coated with RSC3 (5.0 μg/mL) and incubated overnight at 4 °C. Nonfat milk (1% in PBS, 200 µL) was added to each well and incubated for 1 h at 37 °C. Several dilutions of ^89^Zr-VRC01 (3 to 50 μg/mL) in triplicate were added to the wells and incubated for 1 h at 37 °C. A solution of 1 M NaOH was used to solubilize and recover the ^89^Zr-VRC01 bound to RSC3. Each well was washed in triplicate with 0.05% Tween-20 in PBS between steps. The radioactivity associated with the binding of ^89^Zr-VRC01 to RSC3 is counted in an automatic gamma counter (Hidex, Turku, Finland). The dissociation constant (Kd) is calculated using Graphpad Prism 8.0 (La Jolla, CA) by fitting the curve to one-site specific binding.

### Pharmacokinetics and dosimetry in healthy mice using µPET/CT

^89^Zr-DFO-VRC01 dose calculations were performed using temporal imaging data from male and female Balb/C mice (five females and five males) approximately 6–8 weeks old. All studies were carried out under a protocol reviewed and approved by the UCSF IACUC. Mice were allowed to have natural sleep/wake cycles and feeding at will. ^89^Zr-VRC01 (165 ± 3 μCi) was administrated as intravenous bolus injections into a catheterized tail vein, followed by a saline flush. Multiple time-point PET imaging (InVeon, Siemens Nashville, TN) measuring the temporal distribution of the radiotracer ^89^Zr-VRC01 were acquired at 1, 4, 24, 48, and 120 h of post-injection. PET data were acquired in list-mode, histogrammed, and reconstructed using 2D ordered subset expectation maximization (OSEM) algorithm that includes CT-based attenuation correction, and quantification calibration to convert the raw reconstructed pixel value to a physical unit of Bq/mL (OLINDA/EXM vs. 2). Volumes of interest (VOIs) were drawn co-registered CT images for brain, lungs, heart, liver, kidneys, and urinary bladder. All VOIs were either elliptical cylinder (4 mm long axis, 3 mm short axis, and 5 mm height for brain) or cylinders (3 mm diameter and 3 mm height for lungs, heart, and liver, and 2 mm diameter and 2 mm height for kidneys and urinary bladder), and they were placed well within the anatomical boundaries to minimize spill-over of radioactivity. The mean value (in Bq/mL) in these VOIs were multiplied by standard mouse organ volumes (in mL) to derive total activity within these organs. The total activity within the animal subtracted by all organ activities was used as activity in the remainder of the body. Percent of injected activity within the defined organs (%IA) was extrapolated to human-equivalent values using ratios of standard human organ weight to mouse organ weight. Time activity curves were created and the area under the time activity curve was used to estimate the organ dosimetry of ^89^Zr-VRC01 using OLINDA 1.1 (ICRP60).

### Preparation of clinical ^89^Zr-VRC01

Clinical batches of ^89^Zr-VRC01 were prepared in the UCSF Radiopharmaceutical Facility. following the established standard operation procedures. Final product that met all quality control specifications established in the IND were released for administration. The following tests were performed in order to release each batch: appearance, radioactive concentration, final pH, radiochemical ID (SE-HPLC), radiochemical purity (iTLC), filter integrity and bacterial endotoxin. Sterility and binding assays were performed as post release tests.

### HIV envelope sequencing and neutralization testing

Near full-length envelope Sanger sequencing was performed in detail from cell-associated HIV DNA obtained from HIV-infected participants as we have previously described^50,51^. Briefly, consensus sequences were generated for the gp120 loop D, the CD4 binding loop, and the V5 region of the HIV-1 gp120 envelope protein. Ambiguous nucleotides were called if minority peaks from sequencing chromatograms were 30% or greater in height from the main peak. Neutralization capacity to VRC01 was performed on participant samples using the PhenoSense mAb test as previously described using participant-derived pseudoviruses (Monogram Biosciences)^18^.

### PET/MR Imaging

Following study entry and laboratory screening, participants were intravenously injected with ^89^Zr-VRC01 (37 MBq, 1.0 mCi) and PET/MR scans (GE SIGNA) were acquired at 2–3, 4–6, 24, and 72 h over six bed positions (top of head to mid thighs) at 5–10 min/bed position. A subset of ART-suppressed, viremic and uninfected participants were imaged 144 h of post-injection.

### PET image analysis

Standardized uptake values (SUV) in various regions of interest (ROI) from PET-MR data were determined using the OsiriX DICOM viewer software package (Pixmeo; Bernex Switzerland). With the exception of gut tissue, 3-dimensional spherical ROIs were drawn on tissues of interest on axial LAVA MR images (water, fat or in-phase only images) prior to overlaying MR-attenuated PET data to decrease potential analysis and ROI gating bias. Gut tissue was gated on coronal PET-MR reconstructions in order to avoid interferences from intraluminal signal. Following ROI placement (e.g., individual lymph nodes, aortic outflow tract for blood pool, liver, etc.) attenuation corrected PET data were superimposed on MR images and SUV max and mean values were calculated for each ROI. For axillary and inguinal lymph node gating, the largest nodes on both the right and left side were included, and SUV values averaged from the primary analyses. Gating was performed blinded to study group. Two- and three-dimensional PET or PET-MR images were generated in OsiriX keeping PET window levels consistent between participants.

### HIV-flow

The frequency of CD4+ T cells producing the HIV protein p24 was measured by HIV-Flow, as previously described (Pardons et al.). Briefly, CD4+ T cells were isolated from LNMCs by negative magnetic selection (StemCell) and directly stained with the Aqua Live/Dead staining kit and with antibodies against cell-surface molecules in PBS+ 4% human serum for 30 min at 4 °C. Cells were fixed/permeabilized with the FoxP3 Staining Buffer Set (eBioscience) for 45 min and stained with anti-p24 KC57 and anti-p24 28B7 antibodies for an additional 45 min in the permeabilization buffer. The frequency of p24 double positive cells (KC57+, 28B7+) was determined by flow cytometry in gated viable CD8-CD45RA- T cells using FlowJo V10.6. In all experiments, CD4+ T cells from an HIV-uninfected control was included to set the threshold of positivity. A detailed laboratory protocol describing all steps of the HIV-Flow procedure can be accessed here: 10.17504/protocols.io.w4efgte. Titrations were performed for all antibodies to determine optimal concentrations. Dilution used in this study are indicated below:

CD3 UCHT-1 A700 (reference 557943, dilution 1/100), CD4 SK3 BUV496 (reference 564651, dilution 1/25), CD8 RPA-T8 BUV395 (reference 563795, dilution 1/100), CD45RA HI100 BV786 (reference 563870, dilution 1/100), CXCR5 RF8B2 BB515 (reference 564624, dilution 1/50), PD-1 EH12.1 BUV737 (reference 565299, dilution 1/100) were purchased from BD Biosciences. ICOS C398.4A BV421 (reference 313523, dilution 1/100) was obtained from Biolegend. p24 KC57-FITC (dilution 1/1000) was obtained from Beckman Coulter. p24 28B7-APC (dilution 1/1000) was obtained from Medimabs.

### Statistical analyses

We included five participants in each study group (viremic, ART, control) based on the prior SIV PET imaging study^6^ that observed significant differences between four SIV-infected viremic macaques and 2–3 control macaques in various lymphoid tissue ROIs. Blood-pool adjusted rSUVmax and rSUVmean were compared across treatment or infection groups using either Mann–Whitney non-parametric tests (for two groups) or Kruskal–Wallis non-parametric tests for analyses involving three groups. Uncorrected Dunn tests for multiple comparisons were incorporated given the small samples size. Descriptive only statistics were used if there were fewer than four individuals within any comparator group. Spearman rank correlation analyses were used to determine strength and significance of correlations between rSUV and variables such as CD4+ T cell p24 frequencies or time on ART.

### Reporting summary

Further information on research design is available in the Nature Research Reporting Summary linked to this article.

## Supplementary information

Supplementary Information

Reporting Summary

## Data Availability

All data associated with this study are available in the main text or the supplementary materials and Source Data files. Source data are provided with this paper.

## Source data

Source Data
