# Supplementary material for: First-in-human immunoPET imaging of HIV-1 infection using 89Zr-labeled VRC01 broadly neutralizing antibody
Source: Nat Commun. 2022 Mar 9;13:1219. doi: 10.1038/s41467-022-28727-5 (PMC8907355; doi:10.1038/s41467-022-28727-5)
Supplement: Supplementary file 1 — Supplementary Information [file 41467_2022_28727_MOESM1_ESM.pdf]

SUPPLEMENTARY MATERIAL

First-in-human ImmunoPET Imaging of HIV-1 Infection using ^{89}Zr-Labeled VRC01 broadly neutralizing antibody

Denis R. Beckford-Vera[1], Robert R. Flavell[1], Youngho Seo[1], Enrique Martinez-Ortiz[2], Maya Aslam[1], Cassandra Thanh[3], Emily Fehrman[2,] Marion Pardons[4], Shreya Kumar[3], Amelia N Deitchman[5], Vahid Ravanfar[1], Brailee Schulte[1], I-Wei Katherine Wu[1], Tony Pan[3], Jacqueline D Reeves[3], Christopher C. Nixon[3], Nikita S. Iyer[3], Leonel Torres[3], Sadie E. Munter[3], Tony Hyunh[1], Christos J. Petropoulos[6], Rebecca Hoh[2], Benjamin L. Franc[7], Lucio Gama[8], Richard A. Koup[8], John R. Mascola[8], Nicolas Chomont[4], Steven G. Deeks[2], Henry F. VanBrocklin[1]*, Timothy J. Henrich[3]* (*contributed equally)

Affiliations

[1] Department of Radiology and Biomedical Imaging, University of California San Francisco, San Francisco, CA, USA

[2] Division of HIV, Infectious Diseases and Global Medicine, University of California San Francisco, San Francisco, CA, USA

[3] Division of Experimental Medicine, University of California San Francisco, CA, USA

[4] Department of Microbiology, Infectiology and Immunology, Centre de Recherche du CHUM, Université de Montréal, Montreal, Quebec, Canada

[5] Department of Clinical Pharmacy, University of California, San Francisco, USA

[6] Monogram Biosciences, Inc., Laboratory Corporation of America, South San Francisco, USA.

[7] Department of Radiology, Stanford University, Palo Alto, CA, USA

[8] Vaccine Research Center, National Institute for Allergy and Infectious Diseases, National Institutes of Health, Bethesda, Maryland, USA

Communicating Authors:

Timothy J. Henrich, MD, MSc
1001 Potrero Avenue, Bldg 3, Room 525A, San Francisco, CA, 94110
Timothy.Henrich@ucsf.edu 628-206-5518 ORCID: 0000-0002-6684-0622

Henry F. VanBrocklin, PhD
185 Berry Street Bldg B, San Francisco CA 94158
Henry.Vanbrocklin@ucsf.edu 415-353-4569 ORCID: 0000-0003-2849-0841

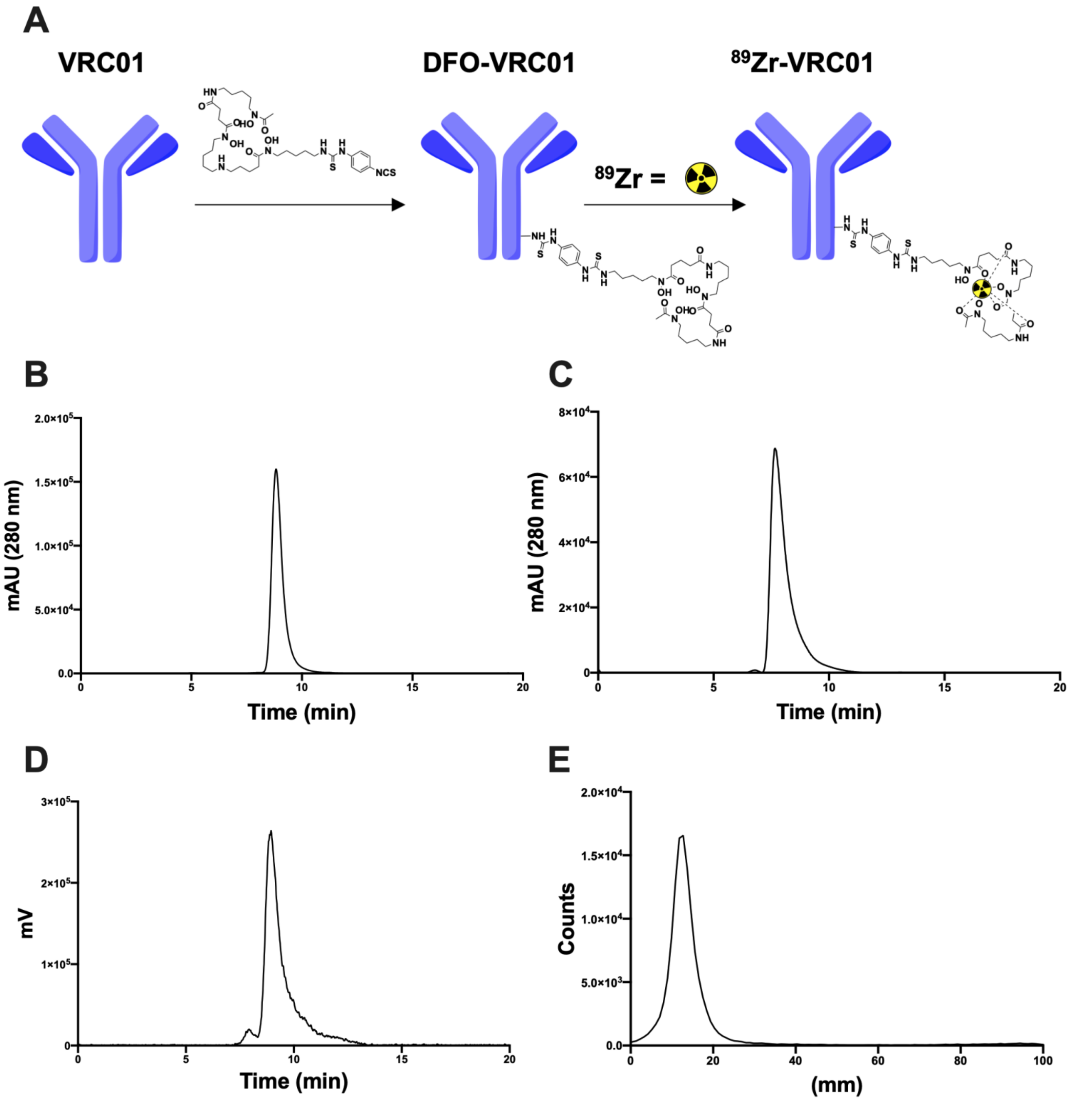

Figure S1. Synthesis and characterization of ^{89}Zr-VRC01. (A) Schematic representation of ^{89}Zr-VRC01 synthesis scheme showing the conjugation of DFO to an amine in the antibody backbone through an activated ester and subsequent chelation of the zirconium-89 to give ^{89}Zr-VRC01. High Performance Liquid Chromatography (HPLC) trace using size exclusion chromatography of (B) unmodified VRC01 (UV @ 280 nm), (C) DFO-VRC01 showing no significant aggregation after conjugation of VRC01 with DFO (UV @ 280 nm) and (D) ^{89}Zr-VRC01 (radioactive detection). (E) Instant thin layer chromatography (iTLC) of purified ^{89}Zr-VRC01 demonstrating that ^{89}Zr-VRC01 was obtained with radiochemical purity higher than 98%.

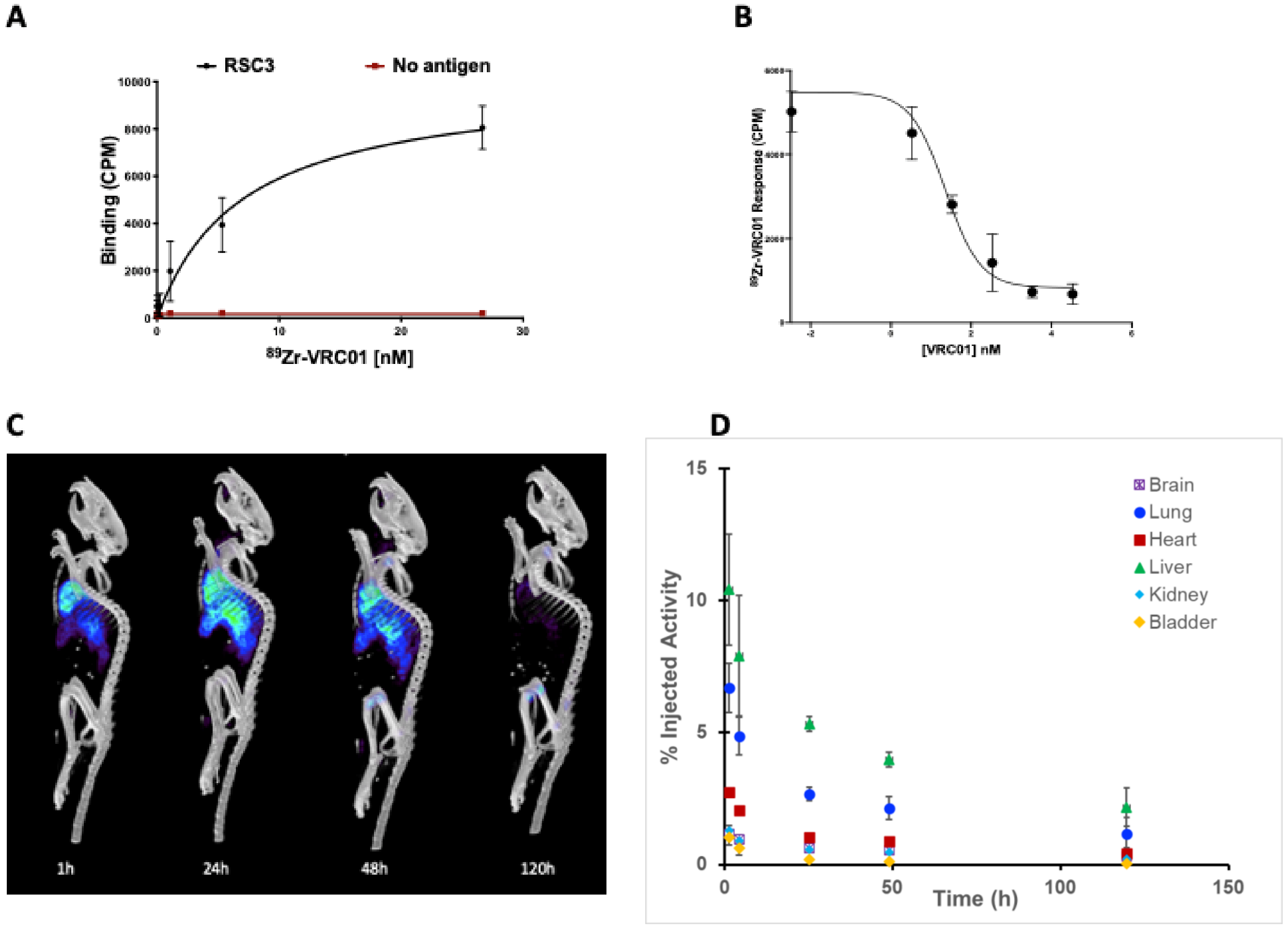

Figure S2. ^{89}Zr-VRC01 binding capacity. **(A)** Binding saturation assay of ^{89}Zr-VRC01 using a resurfaced stabilized core (RSC3) recombinant protein that was used to isolated VRC01.[1] K_D value for ^{89}Zr-VRC01 is 5.21 ± 0.84 (n = 3, R^2 = 0.9232), comparable to the values reported using surface plasma resonance (K_D = 5.62 nM).[2] (B) Binding competition assay using unmodified VRC01 to compete with ^{89}Zr-VRC01 for binding to RSC3 (IC_{50} = 22.7 nM), demonstrating specific binding of ^{89}Zr-VRC01 to RSC3. (C) Pharmacokinetic study of ^{89}Zr-VRC01 in healthy Balb-C mice (5 males/5 females) by small animal Positron Emission Tomography/ Computed Tomography (PET/CT) imaging following injection of 150 µCi (5.55 MBq) ^{89}Zr-VRC01. (D) Tissue/Organ time activity curves of ^{89}Zr-VRC01 derived from the pharmacokinetics study. Error bars represent mean and standard deviation. Source data are provided as a Source Data file.

Table S1. ^{89}Zr-VRC01 Absorbed dose (mGy/MBq) obtained from the early study human participants (N=6) compared to the extrapolated values estimated from the preclinical pharmacokinetic studies in mice (shown in supplementary Figure 2C and 2D).

**Organ**	Human data (mGy/MBq)			Extrapolated from murine data (mGy/MBq)		
Adrenals	0.661	±	0.136	0.360	±	0.005
Brain	0.084	±	0.014	0.149	±	0.006
Breasts	0.252	±	0.044	0.227	±	0.003
Gallbladder Wall	0.986	±	0.226	0.391	±	0.008
LLI Wall	0.258	±	0.048	0.325	±	0.004
Small Intestine	0.356	±	0.070	0.342	±	0.004
Stomach Wall	0.381	±	0.073	0.325	±	0.004
ULI Wall	0.415	±	0.085	0.339	±	0.004
Heart Wall	0.807	±	0.110	0.443	±	0.005
Kidneys	1.005	±	0.200	0.393	±	0.010
Liver	2.905	±	0.725	0.514	±	0.026
Lungs	0.527	±	0.095	0.424	±	0.011
Muscle	0.271	±	0.051	0.269	±	0.003
Pancreas	0.586	±	0.119	0.369	±	0.005
Red Marrow	0.293	±	0.055	0.273	±	0.003
Osteogenic Cells	0.333	±	0.063	0.376	±	0.005
Skin	0.186	±	0.035	0.193	±	0.003
Spleen	0.326	±	0.060	0.313	±	0.004
Testes	0.191	±	0.036	0.257	±	0.003
Thymus	0.332	±	0.051	0.307	±	0.003
Thyroid	0.213	±	0.039	0.275	±	0.003
Urinary Bladder Wall	0.296	±	0.038	0.332	±	0.002
Total Body	0.343	±	0.067	0.278	±	0.003
**Effective Dose (mSv/MBq)**	**0.443**	±	**0.085**	**0.318**	±	**0.004**

Table S2. ^{89}Zr-VRC01 clinical specifications and acceptance criteria for pre-release and post release tests of agent quality. The test results are shown for the validation runs and first clinical batches (two last columns on the right).

Test (Test Method)	Acceptance Criteria	180228ZRVRC-1	180228ZRVRC-2	180307ZRVRC-1	180307ZRVRC-2	181127ZRVRC-1	181211ZRVRC-2
Radiochemical purity (rTLC)	≥90%	99.1 %	99.3%	99.2%	100%	100%	100%
	Monomer: ≥80%	Monomer: 92.3%	Monomer: 95.6%	Monomer: 98.2%	Monomer: 92.7%	Monomer: 98.7%	Monomer: 92.4%
Radiochemical purity (rad-SEC-HPLC)	LMWS: ≤10%	LMWS: 5.2%	LMWS: 1.7%	LMWS: 0.0%	LMWS: 5.8%	LMWS: 0.3%	LMWS: 0.0%
	HMWS: ≤10%	HMWS: 2.3%	HMWS: 2.6%	HMWS: 1.7%	HMWS: 1.4%	HMWS: 0.92%	HMWS: 7.5%
Appearance (Visual observation)	Clear and particle free	Clear and particle free	Clear and particle free	Clear and particle free	Clear and particle free	Clear and particle free	Clear and particle free
pH (pH paper)	4.0-8.0	5.0	5.0	5.3	5.0	5.5	5.5
Endotoxin content (PTS Endosafe, USP<85>)	<35 EU/mL	<5 EU/mL	<5 EU/mL	<5 EU/mL	<5 EU/mL	<5 EU/mL	<5 EU/mL
Filter integrity (Bubble point)	>50 psi	58 psi	58 psi	60 psi	60 psi	60 psi	62 psi
**Post Release**							
Binding assay	$K_D < 20nM$	6.2 nM	10.0 nM	13.8 nM	17.7 nM	10.2 nM	7.9 nM
Sterility	No signs of bacterial growth	No growth	No growth	No growth	No growth	No growth	No growth

Table S3. HIV-1 Env Loop D, CD4 binding site and hypervariable region 5 amino sequencing from PBMC DNA obtained prior to PET-MR imaging. *Intact sequences were not able to be obtained from ART-suppressed participant A3 who was on ART for the longest duration (approximately 23 years).

ID	Group	Loop D	CD4 Binding Site	Variable Loop 5
V1	Viremic	ENFTDNGKIIIVQL	TQHSGGDPEIVMHSFN	TRDGGNGSENGTQVFRPEGGNMRDN
V2	Viremic	ANFSDNAKTIIVQL	KPSAGGDPEVVMHMFN	TRGDGGNQTNGTETFRPGGGDMRDN
V3	Viremic	DNFSNNAKTIIVQL	NHSSGGDLEVVMHSFN	TRDGGNNNNSMNETFRPGGGDMRDN
V4	Viremic	ENFNMWKNNMVE	KRSSGGDPEIVMHSFI	TRDGGTNESEGNETFRPGGGNMMDN
V5	Viremic	ANFSDNAKTIIVQL	NSSSGGDPEIVMHSFN	THDSGSSVERNNDTETFRPRRRDMKDN
A1	ART	KNLSDNAKTIIVQL	DKSSGGDLEVEMLTFN	VRDGGQKNGTNETFRPGGGNMKDN
A2	ART	ENLTNNAKIIIVHL	NSSSGGDPEIVMHSFN	TRDGGIGNNTSNTETFRPAGGDMRDN
A3	ART	*	*	*
A4	ART	DNFSDNAKTLIVQL	NQSSGGDPEIVMHTFN	TRDGG(N/D)ED(T/A)ATFTPGGGNMKDN
A5	ART	QNISDNTKIIIVQL	NHSSGGDPEIVMHSFN	

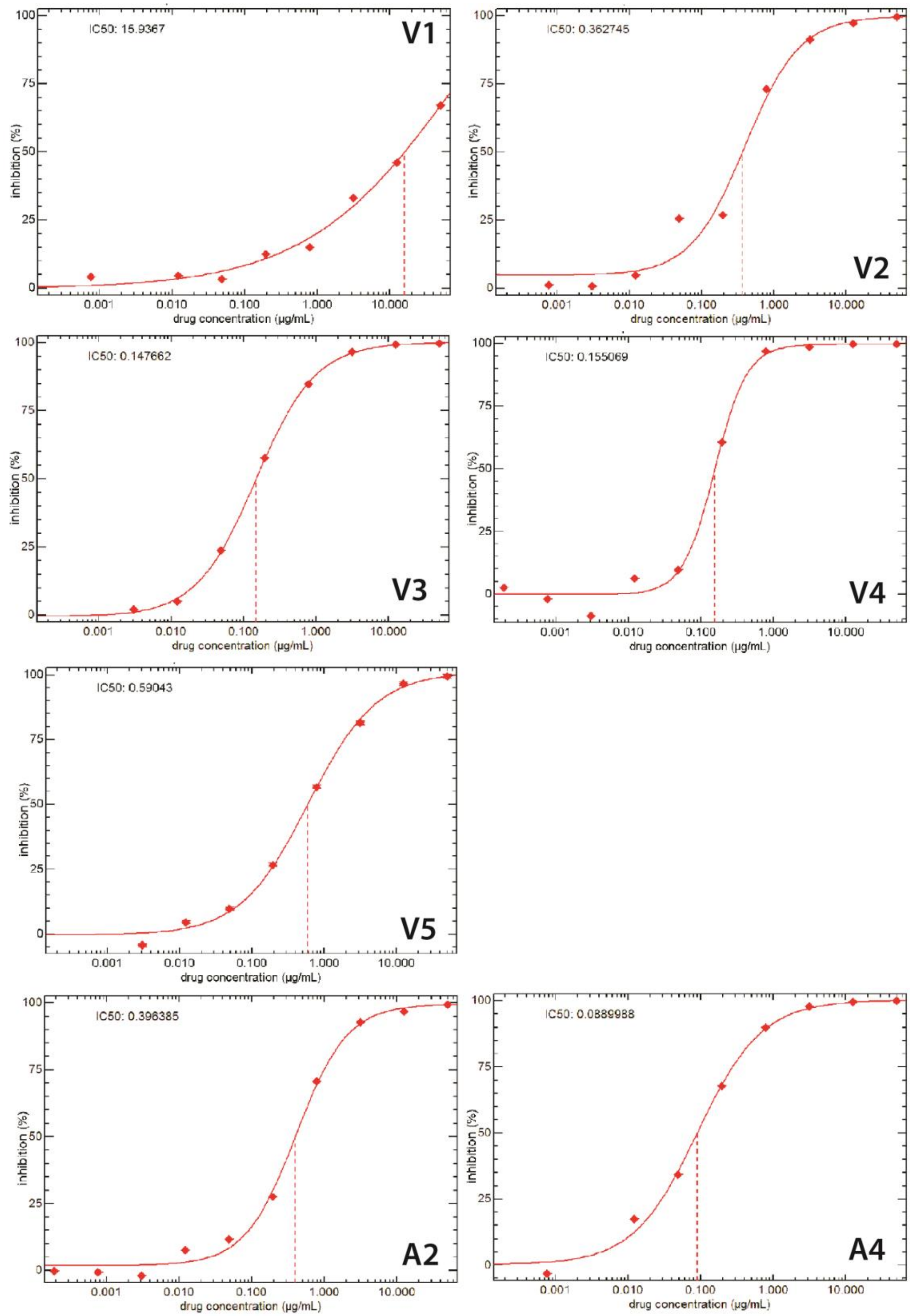

Figure S3. Inhibition of participant-derived pseudoviruses to VRC01 using the Phenosense mAb assay (Monogram Biosciences). IC50 = 50% inhibitory concentration (represented by the dashed red line).

Table S4. Fold Changes in Blood Pool-Adjusted Standardized Uptake Values Between Viremic and ART-Suppressed Participants and Uninfected Controls for Tissue Regions of Interest

Tissue Region of Interest	Imaging Day 0 Fold Change[a] Viremic/Control	Imaging Day 1 Fold Change Viremic/Control	Imaging Day 3 Fold Change Viremic/Control	Imaging Day 3 Fold Change ART/Control
Inguinal LN rSUVmax	**2.35***	3.24‡	**3.04****	1.52
Inguinal LN rSUVmean	**2.47***	3.08‡	**2.52****	**1.99***
Axiallry LN rSUVmax	**2.29***	4.94‡	**3.32****	1.29
Axiallry LN rSUVmean	**2.77***	3.45‡	**2.85****	1.12
Gut (coronal gating) rSUVmax	1.16	1.69‡	1.30	1.09
Gut (coronal gating) rSUVmean	**2.49***	2.60‡	**2.76****	**2.72***
Descending/Sigmoid Colon rSUVmax	**6.39***	4.8‡	**6.4****	**5.44****
Descending/Sigmoid Colon rSUVmean	**6.53***	2.9‡	**3.66****	2.59
Anorectal Junction rSUVmax	1.76	3.27‡	**4.1***	3.54
Anorectal Junction rSUVmax	1.71	2.1‡	**3.6***	1.69
Nasal Turbinates rSUVmax	0.97	1.40‡	**1.53***	**1.55***
Nasal Turbinates rSUVmean	0.80	0.94‡	0.93	1.16
Iliac Marrow rSUVmax	1.06	2.16‡	**1.74***	**1.27***
Iliac Marrow rSUVmean	1.44	1.63	1.57	**1.76***
Femoral Marrow rSUVmax	1.01	1.39‡	**1.76***	1.67
Femoral Marrow rSUVmean	1.59	1.65‡	1.40	**2.92***

rSUV = ratio of SUV between tissue of interest and blood pool

[a] Fold change in blood pool adjusted SUVmax or SUVmean values

* $P < 0.05$ by Mann-Whitney testing of rSUV values between imaging groups

** $P < 0.01$ by Mann-Whitney testing of rSUV values between imaging groups

‡ Statistical analyses not performed as N < 4 (participant V2 did not complete Day 1 imaging)

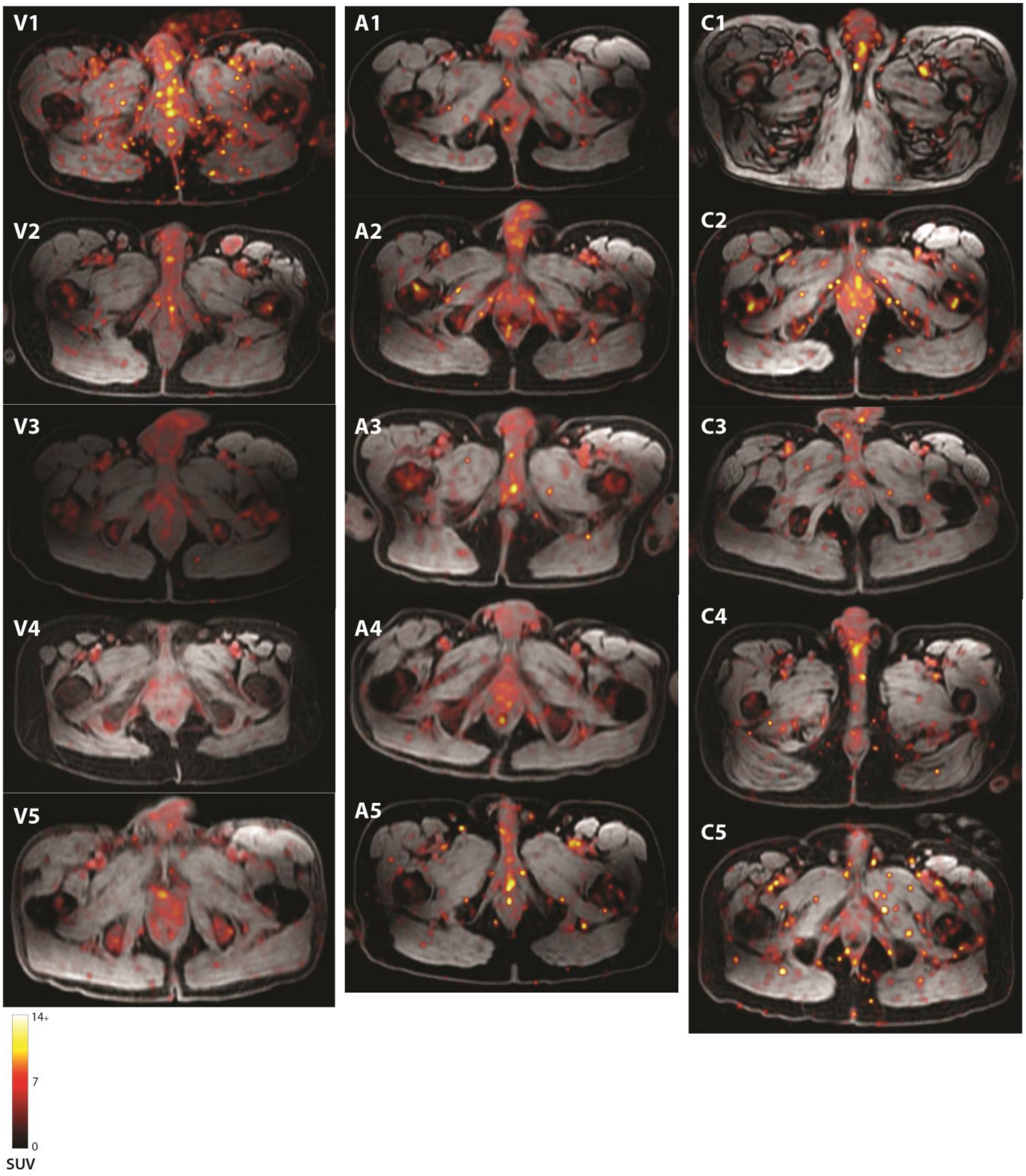

Figure S4. Axial PET-MR images of inguinal lymph node regions from all participants 72 hours following ^{89}ZR-VRC01 administration. V = viremic; A = ART suppressed; C = uninfected control.

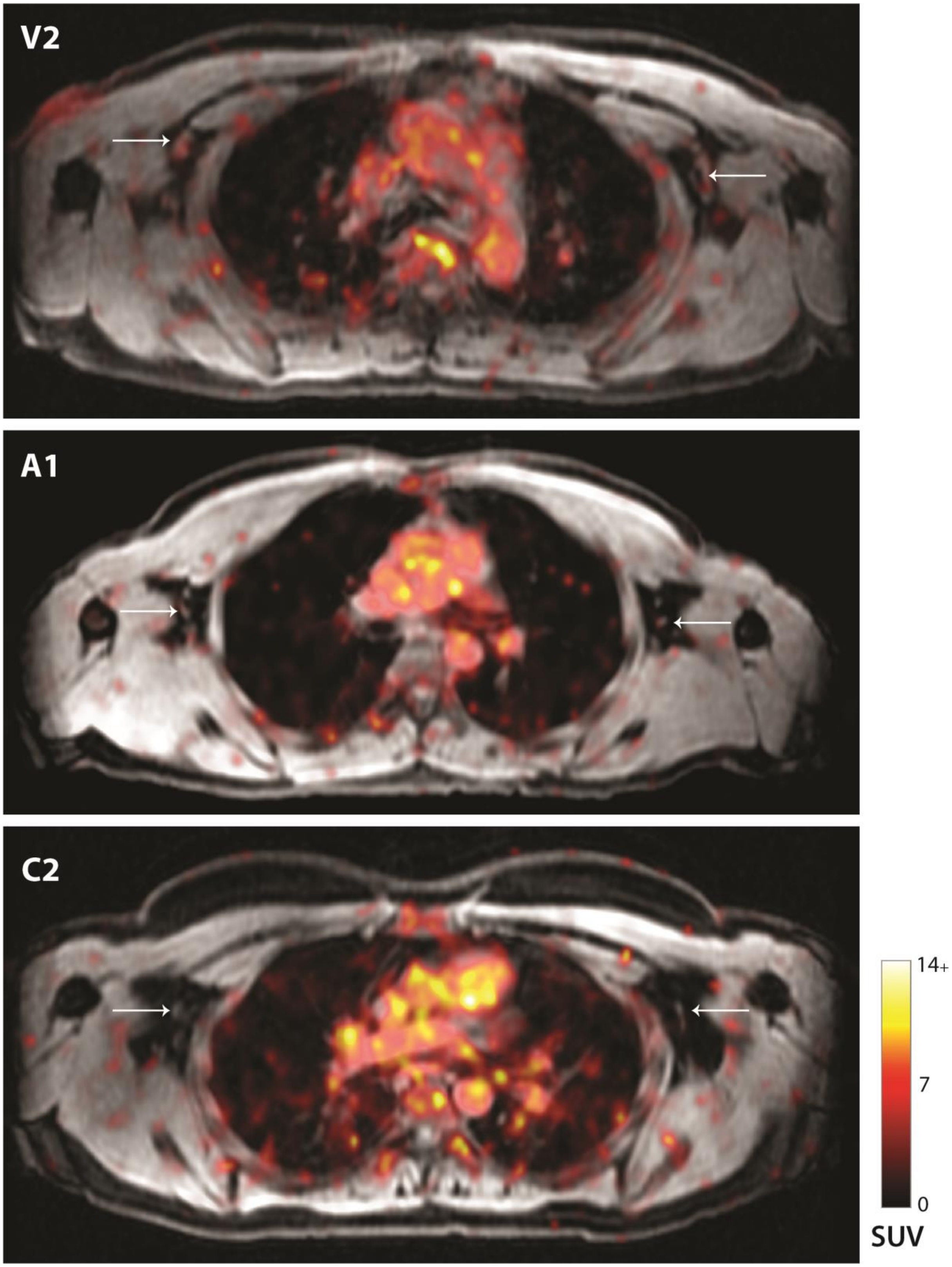

Figure S5. Axial PET-MR images of axillary lymph node regions from representative viremic, ART suppressed and control participants 72 hour following ^{89}ZR-VRC01 administration. White arrows show individual lymph nodes.

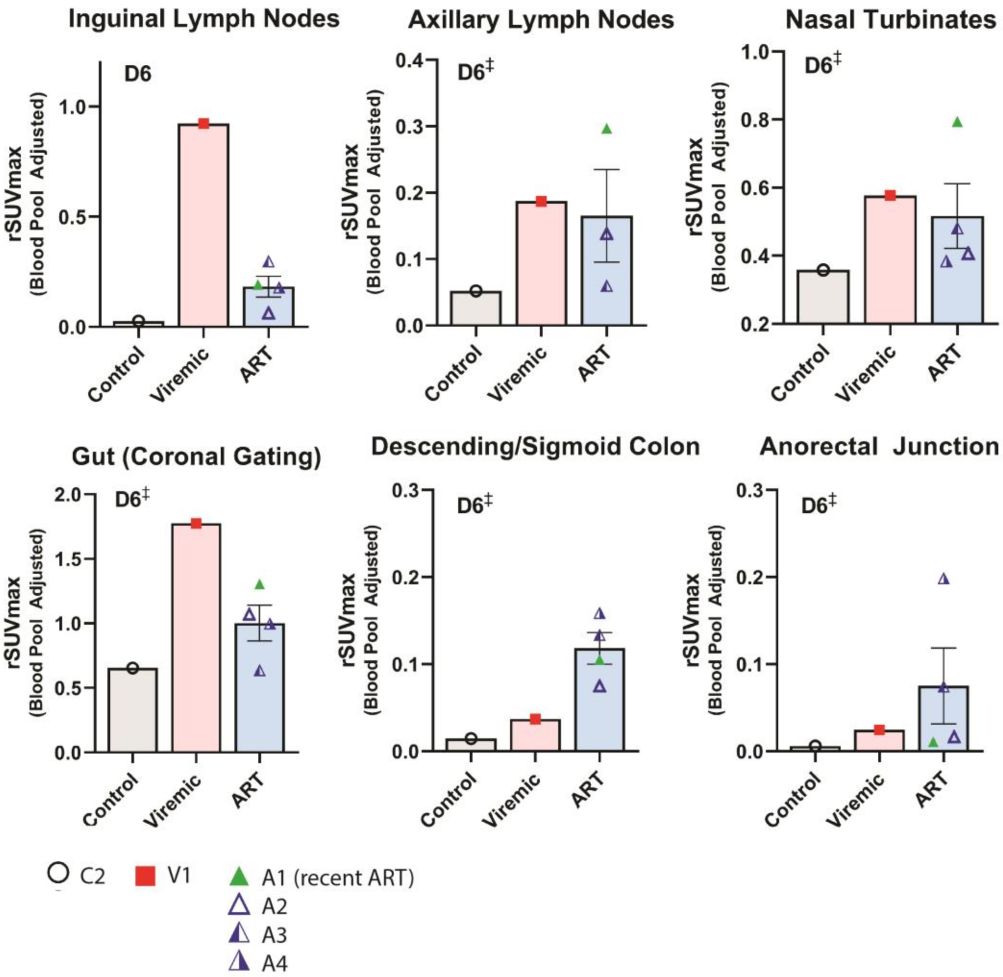

Figure S6. Tracer uptake in tissue 6 days after injection. rSUV max values from inguinal and axillary lymph nodes, nasal turbinates, coronally gated gut tissue, colon and anorectal wall tissue from PET/MR imaging approximately 144 hours following ^{89}ZR-VRC01 administration in an exploratory subset of participants to determine uptake detection over extended periods of time. rSUV = blood pool-adjusted ^{89}Zr-VRC01 standardized uptake value; mean and standard error bars are shown. Control N = 1, viremic N = 1, ART N = 4. Source data are provided as a Source Data file.

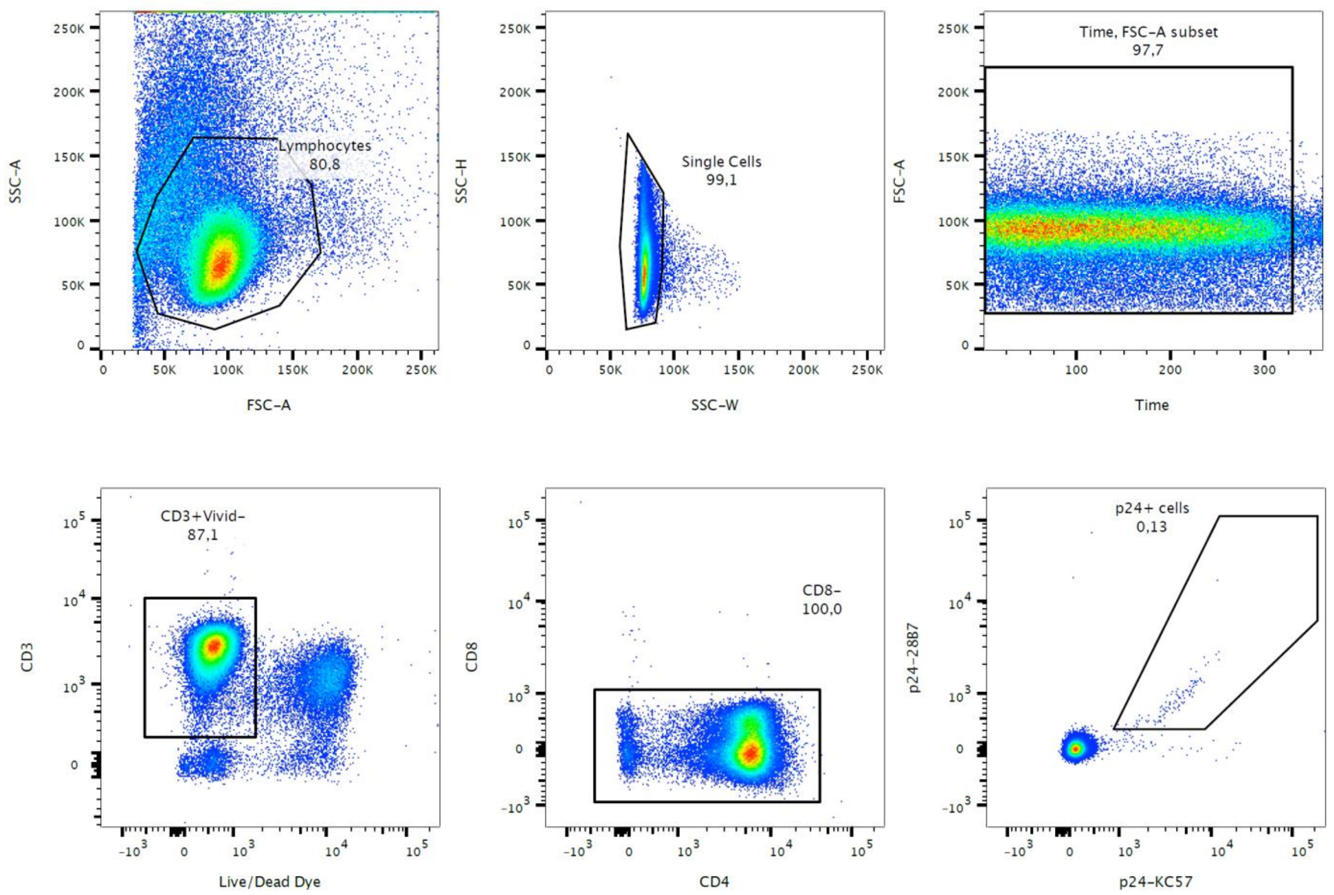

Figure S7. Flow cytometry gating. Example of flow cytometric gating of blood and lymph node T cells expressing HIV p24 antigen. Results from the p24 HIV flow assay are presented in Figure 6b of the main manuscript.

Supplementary References:

1. Lynch, R. M. *et al.* The development of CD4 binding site antibodies during HIV-1 infection. *Journal of Virology* 86, 7588–7595 (2012).
2. Wu, X. *et al.* Rational design of envelope identifies broadly neutralizing human monoclonal antibodies to HIV-1. *Science* 329, 856–861 (2010).
