# Supplementary material for: First-in-human immunoPET imaging of HIV-1 infection using 89Zr-labeled VRC01 broadly neutralizing antibody
Source: Nat Commun. 2022 Mar 9;13:1219. doi: 10.1038/s41467-022-28727-5 (PMC8907355; doi:10.1038/s41467-022-28727-5)
Supplement: Supplementary file 2 — Reporting Summary [file 41467_2022_28727_MOESM2_ESM.pdf]

nature portfolio

Corresponding author(s): Timothy Henrich
Henry VanBrocklin

Last updated by author(s): 11/1/2021

Reporting Summary

Nature Portfolio wishes to improve the reproducibility of the work that we publish. This form provides structure for consistency and transparency in reporting. For further information on Nature Portfolio policies, see our Editorial Policies and the Editorial Policy Checklist.

Statistics

For all statistical analyses, confirm that the following items are present in the figure legend, table legend, main text, or Methods section.

n/a	Confirmed	
☐	☒	The exact sample size (n) for each experimental group/condition, given as a discrete number and unit of measurement
☐	☒	A statement on whether measurements were taken from distinct samples or whether the same sample was measured repeatedly
☐	☒	The statistical test(s) used AND whether they are one- or two-sided<br>*Only common tests should be described solely by name; describe more complex techniques in the Methods section.*
☐	☒	A description of all covariates tested
☐	☒	A description of any assumptions or corrections, such as tests of normality and adjustment for multiple comparisons
☐	☒	A full description of the statistical parameters including central tendency (e.g. means) or other basic estimates (e.g. regression coefficient) AND variation (e.g. standard deviation) or associated estimates of uncertainty (e.g. confidence intervals)
☐	☒	For null hypothesis testing, the test statistic (e.g. F, t, r) with confidence intervals, effect sizes, degrees of freedom and P value noted<br>*Give P values as exact values whenever suitable.*
☒	☐	For Bayesian analysis, information on the choice of priors and Markov chain Monte Carlo settings
☒	☐	For hierarchical and complex designs, identification of the appropriate level for tests and full reporting of outcomes
☒	☐	Estimates of effect sizes (e.g. Cohen's d, Pearson's r), indicating how they were calculated

Our web collection on statistics for biologists contains articles on many of the points above.

Software and code

Policy information about availability of computer code

Data collection	Image data were collected using a GE SIGNA PET-MR scanner. Flow cytometric data were acquired on an BD LSRII. PCR data was generated using the ABI StepOne real-time PCR system.
Data analysis	PET image analyses was performed on DICOM files using Osirix (Pixmeo; Bernex Switzerland); Graphical and statistical analyses were performed on GraphPad Prism v.8. Flow cytometric data was analyzed using BS FACS Diva and FlowJo (vs. 10.6). 2D OSEM dosimetry data was analyzed using OLINDA/EXM vs. 2

For manuscripts utilizing custom algorithms or software that are central to the research but not yet described in published literature, software must be made available to editors and reviewers. We strongly encourage code deposition in a community repository (e.g. GitHub). See the Nature Portfolio guidelines for submitting code & software for further information.

Data

Policy information about availability of data

All manuscripts must include a data availability statement. This statement should provide the following information, where applicable:
- Accession codes, unique identifiers, or web links for publicly available datasets
- A description of any restrictions on data availability
- For clinical datasets or third party data, please ensure that the statement adheres to our policy

All data associated with this study are available in the main text or the supplementary materials

Field-specific reporting

Please select the one below that is the best fit for your research. If you are not sure, read the appropriate sections before making your selection.

☒ Life sciences ☐ Behavioural & social sciences ☐ Ecological, evolutionary & environmental sciences

For a reference copy of the document with all sections, see nature.com/documents/nr-reporting-summary-flat.pdf

Life sciences study design

All studies must disclose on these points even when the disclosure is negative.

Sample size	This is an exploratory first-in human PET imaging study and incorporated three comparison groups. The sample size for this study was based on prior non-human primate imaging as described in the manuscript text, that observed differences between tissue PET uptake regions with fewer animals than included in this study (Santangelo PJ et al. Nat Methods, 2015; 12(5):427-32. This study used 4 viremic macaques and 3 controls; significant differences in lymphoid and other tissues were identified using PET imaging with these numbers of animals.
Data exclusions	Data were acquired from the planned tissue regions of interest that were determined prior to final study data analysis. ROIs were excluded if it was not possible to perform adequate tissue gating using the Osirix software and quality of PET-MR data. No other data was excluded.
Replication	Image analysis was performed for each imaging time point (ranging from 2-4) for each of the five individuals from each of the three comparison groups. For axillary and inguinal lymph node analyses, mean SUVs were calculated from two separate lymph nodes on contralateral sides of the body.
Randomization	This was not a randomized clinical trial or case controlled study, but all attempts were made to include participants with similar demographic and clinical factors across the comparison groups. This data is explicitly detailed in the stud manuscript.
Blinding	Raw image files were analyzed (ROI selection, tissue SUV calculation) blinded as to the participant information, including the comparison group. Regions of interest were selected without knowing the HIV status or other personal information. Given the nature of the imaging study, investigators were not blinded as to the selection of participants within each group as they were responsible for enrolling participants and scheduling imaging visits.

Reporting for specific materials, systems and methods

We require information from authors about some types of materials, experimental systems and methods used in many studies. Here, indicate whether each material, system or method listed is relevant to your study. If you are not sure if a list item applies to your research, read the appropriate section before selecting a response.

Materials & experimental systems

n/a	Involved in the study	
☐	☒	Antibodies
☒	☐	Eukaryotic cell lines
☒	☐	Palaeontology and archaeology
☐	☒	Animals and other organisms
☐	☒	Human research participants
☐	☒	Clinical data
☒	☐	Dual use research of concern

Methods

n/a	Involved in the study	
☒	☐	ChIP-seq
☐	☒	Flow cytometry
☒	☐	MRI-based neuroimaging

Antibodies

Antibodies used	p24 KC57-PE (Beckman Coulter, 6604667)· p24 28B7-APC (Medimabs, MM-0289-APC)
Validation	p24 antibodies as above were titrated upon reception of each new batch acquired from the manufacturer. Lot numbers and expiration dates were recorded. Positive controls were used with each flow cytometry experiment.

Animals and other organisms

Policy information about studies involving animals; ARRIVE guidelines recommended for reporting animal research

Laboratory animals	Balb/C mice (5 females and 5 males). Mice were allowed natural sleep/wake cycles and were approximately 6-8 weeks old.
Wild animals	No wild animals were used in this study.
Field-collected samples	No field-collected samples were used in this study.

Ethics oversight	All studies were carried out under a protocol reviewed and approved by the UCSF IACUC

Note that full information on the approval of the study protocol must also be provided in the manuscript.

Human research participants

Policy information about studies involving human research participants

Population characteristics	Fifteen participants 18 years of age or older were recruited for this imaging study, including HIV-infected participants on continuous suppressive ART with viral load < 40 RNA copies/mL prior to imaging and within 12 months of study entry or detectable plasma HIV RNA > 1,000 copies per/mL, and uninfected controls. Viremic individuals had plasma HIV-1 RNA ranging from 3,499 to 789,705 copies/mL) and ART-suppressed individuals had times on ART ranging from 105 to 8,509 days on ART). One viremic and one control participant were female. The mean ages of uninfected control, viremic and ART-suppressed participants were 58, 52 and 53 years, respectively. In addition, HIV+ participants had HIV-1 envelope RNA or DNA consensus sequences from peripheral blood suggestive of VRC01 binding activity (HIV infected participants only) when able. Study procedures were conducted between 2017 and 2019.
Recruitment	Participants were identified from the UCSF SCOPE cohort and HIV clinics at Zuckerberg San Francisco General Hospital between 2017 and 2019. These are outpatient units that provide clinical care or research to people living with HIV in the greater San Francisco-Bay Area. Participants were co-enrolled in the HIV SCOPE study for additional collection of blood and tissue for measures of HIV persistence. To minimize self selection bias, investigators identified potential participants based on ART usage, time on ART and viral load levels (for viremic participants).
Ethics oversight	The University of California San Francisco Committee on Human Research approved this clinical study and all participants provided written informed consent and underwent MRI screening prior to imaging.

Note that full information on the approval of the study protocol must also be provided in the manuscript.

Clinical data

Policy information about clinical studies

All manuscripts should comply with the ICMJEguidelines for publication of clinical research and a completedCONSORT checklist must be included with all submissions.

Clinical trial registration	ClinicalTrials.gov: NCT03729752.
Study protocol	The full study protocol can be obtained from the corresponding authors upon request.
Data collection	Participants were recruited at ZSFGH and PET-MR imaging and data acquisition was performed at the UCSF China Basin radiology/clinical imaging center. Participants provided informed consent and peripheral blood collection at SFGH Research outpatient Ward 84 (SCOPE cohort); PET-MR imaging and tracer injection was performed at the outpatient UCSF Radiology Center at China Basin, San Francisco. Lymph node biopsies were performed at the ZSFG outpatient clinical research ward.
Outcomes	The primary outcome was to observe a difference in rSUV values in lymph node tissues (and other ROIs including gut tissue) between participants with HIV off ART and uninfected controls. Secondary outcomes were to observe differences between those with HIV on ART and uninfected controls.

Flow Cytometry

Plots

Confirm that:

- [x] The axis labels state the marker and fluorochrome used (e.g. CD4-FITC).
- [x] The axis scales are clearly visible. Include numbers along axes only for bottom left plot of group (a 'group' is an analysis of identical markers).
- [x] All plots are contour plots with outliers or pseudocolor plots.
- [x] A numerical value for number of cells or percentage (with statistics) is provided.

Methodology

Sample preparation	CD4+ T cells were isolated from LNMCs by negative magnetic selection (StemCell) and directly stained with the Aqua Live/Dead staining kit and with antibodies against cell-surface molecules in PBS + 4% human serum for 30 min at 4°C. Cells were fixed/permeabilized with the FoxP3 Staining Buffer Set (eBioscience) for 45 min and stained with anti-p24 KC57 and anti-p24 28B7 antibodies for an additional 45 min in the permeabilization buffer. All antibodies are commercially available. Titrations were performed for all antibodies to determine optimal concentrations. Dilution used in this study are indicated below:<br>CD3 UCHT-1 A700 (reference 557943, dilution 1/100), CD4 SK3 BUV496 (reference 564651, dilution 1/25), CD8 RPA-T8 BUV395 (reference 563795, dilution 1/100), CD45RA HI100 BV786 (reference 563870, dilution 1/100), CXCR5 RF8B2 BB515 (reference 564624, dilution 1/50), PD-1 EH12.1 BUV737 (reference 565299, dilution 1/100) were purchased from BD Biosciences. ICOS C398.4A BV421 (reference 313523, dilution 1/100) was obtained from Biolegend. p24 KC57-FITC (dilution 1/1000) was obtained from Beckman Coulter. The certificate of analysis can be found on the Beckman Coulter website. It is certified that each batch of p24 KC57 meets the requirements for flow cytometry experiments.

	p24 28B7-APC (dilution 1/1000) was obtained from Medimabs.
Instrument	BD LSRII
Software	BD FACS Diva for data acquisition, FlowJo v.10 for analysis.
Cell population abundance	In all experiments, CD4+ T cells from an HIV-uninfected control was included to set the threshold of positivity. All cells of interest were collected from each sample.
Gating strategy	The frequency of p24 double positive cells (KC57+, 28B7+) was determined by flow cytometry in gated viable CD8-CD45RA- T cells using FlowJo V10.6. A detailed laboratory protocol describing all steps of the HIV-Flow procedure can be accessed here: dx.doi.org/10.17504/protocols.io.w4efgte.

☒ Tick this box to confirm that a figure exemplifying the gating strategy is provided in the Supplementary Information.
